# Clinical indicators associated with pain trajectories in non-specific neck pain: a secondary analysis of a multicentre prospective observational cohort to develop a clinical compass for personalised physiotherapy in primary care

**DOI:** 10.1136/bmjopen-2025-115274

**Published:** 2026-08-03

**Authors:** Martine J Verwoerd, Richard A W Felius, Sophie Konings, Henri Kiers, Rob J E M Smeets

**Affiliations:** 1 Research Group Clinical Decision Making in Movement Care, Utrecht University of Applied Sciences, Utrecht, Netherlands; 2 Department of Rehabilitation Medicine, Care and Public Health Research Institute (CAPHRI), Maastricht University, Maastricht, Netherlands; 3 CIR Clinics in Revalidatie, Eindhoven, Netherlands; 4 Pain in Motion International Research Group, Maastricht, Netherlands

**Keywords:** PAIN MANAGEMENT, Clinical Reasoning, Musculoskeletal disorders, Prognosis

## Abstract

**Abstract:**

**Objectives:**

To identify clinical indicators reflecting potentially treatment-modifiable prognostic factors associated with pain recovery in patients with non-specific neck pain and to develop a prototype clinical compass for visualising patient-specific profiles.

**Design:**

Secondary analysis of data from a multicentre, prospective, observational prognostic cohort study, with a 6-month follow-up between January 2020 and March 2023.

**Setting:**

30 physiotherapy primary care practices.

**Participants:**

Patients with a new episode of non-specific neck pain.

**Primary and secondary outcome measures:**

Patients completed questionnaires at baseline, 6 weeks, 3 months and 6 months assessing pain intensity and biological, psychological and social factors. Questionnaire total scores and individual items reflecting selected potentially modifiable factors were tested separately as candidate indicators. Baseline and repeated indicators were examined as effect modifiers of pain trajectories using growth-curve models. Subsequently, modifiable clinical indicators were incorporated as effect modifiers to evaluate their influence on the trajectory of pain intensity over time. Non-redundant significant indicators were integrated into a prototype clinical compass.

**Results:**

Data from 603 participants were collected. A quadratic growth-curve model best explained the longitudinal change of pain intensity. 24 clinical indicators were significantly associated with the trajectory of pain intensity over time. Two characteristics were excluded as they demonstrated high intercorrelation. Based on these findings, a clinical compass comprising 22 clinical indicators was developed to visualise patient-specific profiles.

**Conclusions:**

This study provides a prototype clinical compass that may inform prognostic reasoning in primary care physiotherapy, but further evaluation is needed before clinical implementation.

**STRENGTHS AND LIMITATIONS OF THIS STUDY**

- Longitudinal design with repeated Numeric Pain Rating Scale (NPRS) measurements, allowing modelling of pain trajectories over time.
- Broad, data-driven candidate set restricted to prespecified, clinically plausible domains, using both total scores and item-level indicators.
- NPRS was collected without a specified recall period, which may have increased measurement error and attenuated associations.
- The large, data-driven candidate set entails risks of type I and type II error, and no causal effects of indicator changes on pain trajectories were estimated.

## Introduction

 The vast majority of neck pain cases are non-specific; only a very small proportion reflects serious specific pathology.^1^ Neck pain represents a substantial societal and economic burden; costs related to neck pain are rising mainly due to extended work absences and the use of healthcare services.^2–4^ Non-specific neck pain (NSNP) is recognised as a multifactorial condition influenced by biological, psychological and social factors.^5 6^ Personalised approaches based on comprehensive biopsychosocial assessment hold promise for improving both patient outcomes and healthcare efficiency.^7–9^

In routine primary care, the heterogeneity of NSNP complicates the implementation of such personalised approaches. A broad range of prognostic factors, including psychological distress, pain behaviour, sleep quality and social context, may be relevant to assessment and targeted treatment.^10–16^ Early identification of potentially modifiable prognostic factors may support timely and individualised management and may help reduce the risk of persistent pain. Existing prognostic models, including those developed using this cohort, have provided valuable insights into recovery patterns in NSNP.^14 16^ However, these models often rely on lengthy questionnaires, which limits their practicality in routine clinical care. Stratification based on a concise set of prognostic screening questions offers a feasible alternative to lengthy instruments and may better support personalised care.^10^ For example, existing tools such as the STarT Back Screening Tool have shown promise in managing low back pain by classifying patients into high-risk, medium-risk and low-risk groups. In NSNP, however, findings have been mixed, providing only broad risk categories that offer limited guidance for personalised treatment planning.^7^

Potentially modifiable prognostic factors are defined here as patient characteristics that may be amenable to change through treatment and associated with subsequent pain trajectories. The present study examines whether selected single-item clinical indicators from validated questionnaires can function as pragmatic signals of potentially relevant modifiable domains in routine primary care. These indicators are intended to support focused assessment and prognostic reasoning, while full questionnaires remain relevant for measuring broader underlying constructs. Moving beyond broad stratification towards a more personalised and data-driven approach may therefore enhance the clinical usefulness of prognostic information in primary care physiotherapy. A concise clinical compass based on empirically selected clinical indicators may support patient-specific profiling while remaining feasible in routine care. Examining combinations of clinical indicators, rather than isolated factors alone, may provide a more informative basis for prognostic reasoning and individualised assessment.

This analysis extends previous work from the same cohort, which focused on developing a multivariable prognostic model for chronic pain as a dichotomous outcome.^14^ The present study takes a more practice-oriented, trajectory-based approach by examining questionnaire-level and item-level clinical indicators of pain intensity over time and translating nonredundant indicators into a patient-specific visual profile. Pain intensity was selected as the outcome because it was the primary variable assessed repeatedly across follow-up in the original cohort, allowing recovery to be modelled as a dynamic process over time.

This study aimed to develop a data-driven clinical compass to support prognostic reasoning, patient-specific assessment and a more personalised approach to care for patients with acute or subacute NSNP in primary care physiotherapy. The objectives were to (1) identify clinical indicators of modifiable prognostic factors associated with recovery trajectories in NSNP and (2) compose a patient-specific profile by visualising significant clinical indicators of contributing modifiable prognostic factors to support individualised clinical reasoning.

## Methods

### Study design

This study is a secondary analysis of data from a multicentre, prospective, observational prognostic cohort conducted in primary care physiotherapy, with repeated assessments at four time points.

#### Study setting

Data were collected from 30 primary care physiotherapy practices, involving 81 physiotherapists. Participant recruitment took place between 26 January 2020 and 31 August 2022. The data collection process, including follow-up reminders and response time, was completed by 17 March 2023.

To enhance generalisability, participants were recruited by physiotherapists with diverse professional backgrounds, including those pursuing master’s degrees, experienced clinicians, and practitioners affiliated and unaffiliated with academic institutions.

#### Patient and public involvement

Patients or the public were not involved in the design, or conduct, or reporting, or dissemination plans of our research.

### Participants

Patients were eligible for inclusion if they presented with a new episode of (sub)acute (0–12 weeks) NSNP. Inclusion criteria were: (1) age 18 years or older; (2) symptom onset within the past 12 weeks; (3) pain localised in the area between the occiput and spine of the scapula^17^ and (4) if previously affected by neck pain, a symptom-free period of at least 3 months (Numeric Pain Rating Scale, NPRS <3) prior to the current episode.

Exclusion criteria were assessed during the initial consultation. They included prior neck surgery, cervical radiculopathy (assessed by a negative Upper Limb Neurodynamic Test 1),^18^ widespread pain (as defined by International Classification of Diseases-11),^19 20^ non-musculoskeletal causes of pain^21^ and inability to read or understand Dutch. Physiotherapists recorded reasons for exclusion and maintained anonymised logs of eligible patients who declined participation, including their reasons.

### Baseline and follow-up procedure

Eligible patients were informed about the study during their first consultation. On verbal agreement, written informed consent was obtained before completing the baseline questionnaire. Participants received digital questionnaires via email at baseline (T0), 6 weeks (T1), 3 months (T2) and 6 months (T3) by their treating physiotherapist. The T0 questionnaire required approximately 30–40 min to complete, while T1, T2 and T3 each required 20–30 min. If a questionnaire was not completed within 1 week, a reminder was sent via email or the physiotherapist contacted the patient by phone. This procedure was repeated once if necessary. The responses were accessible only to the research team and were not shared with the treating physiotherapists.

Most potential prognostic factors and the outcome variable (pain) were assessed at each time point, whereas baseline characteristics were collected only at T0.

### Outcome

Pain intensity was assessed using the NPRS (0–10), ranging from 0=no pain to 10=worst imaginable pain, based on the question: ‘How much pain do you experience in your neck?’.^22^

### Factors

The baseline measured variables were selected for their potential prognostic value based on a prior systematic review^23^ and a Delphi study^24^ among neck pain experts. Modifiable factors formed the analytic candidate set; non-modifiable descriptors were collected to characterise the sample and were not eligible for selection in the present study.

#### Non-modifiable descriptive set

- Patient characteristics: sex and age.
- Symptom-related: symptom duration (in weeks).

#### Modifiable factors included for analysis

We analysed all questionnaire total scores and all individual items from each administered questionnaire as clinical indicators.

- Symptom-related: pain intensity at baseline (NPRS),^22^ presence of pain in other body regions (yes/no), accompanying headache (either concurrent with or preceding neck pain) and disability (Pain Disability Index, averaged across completed items).^25^
- Work-related: job satisfaction, perceived happiness at work and ability to self-modify posture (self-reported).^24^
- Lifestyle: smoking status, alcohol use, body mass index, sleep quality (adjusted item from the Neck Disability Index)^26^ and physical activity, determined by whether participants met the Dutch Healthy Exercise Norm (yes/no).^27^
- Psychological and behavioural: illness perceptions (Brief Illness Perception Questionnaire),^28^ pain catastrophising (Pain Catastrophising Scale, short version),^29^ depression and distress (Depression Anxiety Stress Scale-21), kinesiophobia (Tampa Scale for Kinesiophobia, 11-item version),^30^ coping strategies (Pain Coping Inventory, PCI),^31^ hypervigilance (Pain Vigilance and Awareness Questionnaire)^32 33^ and pain self-efficacy (2-item Pain Self-Efficacy Questionnaire).
- Therapeutic context: self-reported trust in the physiotherapist after the first consultation.^34^

Further details on all variables and measurement instruments are available in the published study protocol.^35^

### Statistics

The complete dataset and Python script used in this study are available on GitHub at https://github.com/RichardFel/Paincare.^36^ Growth curve models were used to estimate the longitudinal change in pain intensity and to identify clinical indicators that influenced pain trajectories. Growth curve models can estimate both between-person and within-person differences and are therefore particularly suited to model change over time.^37^ The models were implemented with linear mixed models (LMMs) using Python (V.3.12.7) and the Statsmodels package (V.0.14.4). LMMs incorporate both fixed effects, representing average changes across individuals, and random effects, which account for individual variability. They also allow the inclusion of all available repeated observations, so that participants with incomplete follow-up data can be retained under the assumption that data are missing at random.

#### Longitudinal change in pain intensity

First, the longitudinal change in pain intensity, measured across four time points (0, 6, 12 and 26 weeks), was estimated using a univariate growth-curve model. To account for the correlation of repeated measures within individuals, a random intercept at the participant level was included. Next, a quadratic time term was added to assess whether a non-linear trajectory was a better fit than a linear model. The log-likelihood ratio test (LLRT) was used to compare the fit of the two models, of which the best was selected as the baseline model.

#### Modifiable clinical indicators influencing pain trajectories

To determine whether the modifiable clinical indicators influenced the longitudinal change in pain intensity, they were added separately as effect modifiers by including an interaction term with time (factor × time) in the baseline model (2.6.1). This approach was used as an exploratory screening strategy aimed at identifying which individual indicators are independently associated with pain trajectories, rather than constructing a fully adjusted multivariable prediction model. Consequently, interrelationships between predictors were not modelled simultaneously. A significant outcome (p<0.05) indicates that the clinical indicators influence the trajectory of pain intensity. No correction for multiple testing was applied, since all the included variables were pre-selected based on theoretical and clinical relevance to pain trajectories, which constrains the risk of spurious findings, and the analysis was mainly exploratory. Additionally, applying stringent multiplicity corrections (eg, Bonferroni) in this context would substantially reduce statistical power given the moderate sample size, increasing the risk of excluding clinically important effect modifiers (type II error). Some factors were only measured at baseline, while others were measured repeatedly. For the clinical indicators with repeated measurements, data at all time points were included. Additionally, the marginal R² is calculated to indicate the proportion of variance in pain intensity that can be attributed to the modifiable clinical indicators, independent of the random participant-level structure. It is derived by decomposing total model variance into fixed-effect variance, random-effect variance, and residual variance, following the method described by Nakagawa and Schielzeth.^38^ The marginal R² thus reflects only the contribution of the fixed effects, making it appropriate for comparing the relative explanatory value of each clinical indicator.

#### Collinearity among clinical indicators

Collinearity among clinical indicators was assessed using Pearson’s correlation coefficient. For variables that were repeatedly measured, correlations were calculated across all available time points. For variables assessed only at baseline, correlations were necessarily computed at that single time point; these mainly included stable demographic and clinical characteristics (eg, age, sex, complaint duration) that were not expected to change during follow-up. Clinical indicators showing overlap (defined a priori as) were consolidated or removed to reduce redundancy and respondent burden, thereby reducing the risk that the compass contains multiple indicators conveying the same information, and ensuring that the clinical compass remained applicable and feasible for routine clinical use.^39 40^ Where pairs of significant indicators exceeded this threshold, the retained indicator was selected based on predefined practical and clinical criteria, including strength of association with changes in pain over time, clinical actionability within physiotherapy practice, and non-redundancy in content. Where content overlapped, more specific and informative items were preferred over broader composite scores.

#### Visualisation of the factors for clinical use

The remaining clinical indicators were incorporated into the Clinical Compass radar chart to facilitate visualisation of patient-specific results. This chart provides a multidimensional representation of an individual’s profile relative to the group, allowing inspection of multiple clinical indicators. The clinical indicators were ranked on relevance by using the marginal R^2^. To enable direct comparability across domains, all values were standardised to Z-scores, allowing comparison of an individual’s scores relative to the distribution of other participants. For most included metrics, which are individual items or subscales from questionnaires, no established clinically anchored thresholds exist; sample-based standardisation therefore provides a pragmatic and transparent alternative. Scores above or below zero indicate whether an individual’s outcome lies above or below the group mean, while the magnitude of deviation reflects the distance from the average. This visualisation allows patients and therapists to quickly identify patient-specific factors that may contribute positively or negatively to outcomes.

## Results

### Participants

Of the 2567 patients assessed for eligibility across 30 physiotherapy primary care practices, 660 provided informed consent, and 603 completed the baseline assessment and were included in this study. The main reasons for exclusion were pre-existing chronic pain, cervical spine radiculopathy, and widespread pain; 307 eligible patients declined participation. At follow-up, data were available for 449 participants at 6 weeks, 379 at 3 months and 391 at 6 months. Only 78 participants did not complete any follow-up measurement. Participant flow is presented in online supplemental appendix 1, participant characteristics in table 1, and data availability per variable and measurement occasion in online supplemental appendix 2.

**Table 1:** Patient characteristics and clinical indicators

		Units	0 weeks	6 weeks	12 weeks	26 weeks
Patient characteristics	Age	μ±σ	44±16	–	–	–
	Sex	M/F	206/397	–	–	–
	Duration	μ±σ	4.6±3.0	–	–	–
	Recurrent	Yes/No	198/404	–	–	–
	Education	μ±σ	6±1.7	–	–	–
	BMI	μ±σ	25.3±4.3	–	–	–
Patient-reported outcome measures	Pain intensity	μ±σ	5.9±1.9	2.6±2.6	2.0±2.6	1.5±2.6
	SOM PDI	μ±σ	18.6±14.0	14.0±12.7	NM	NM
	SOM PCS	μ±σ	4.6±4.6	3.8±4.0	4.1±4.5	3.7±4.4
	SOM TSK	μ±σ	16.5±5.2	15.7±4.5	NM	NM
	SOM PSEQ	μ±σ	10.2±2.3	6.6±5.3	10.5±1.9	10.0±2.7
	SOM PVAQ	μ±σ	31.0±11.4	27.5±12.3	27.4±12.5	25.1±13.8
	SOM DASS-21 Dep	μ±σ	2.5±3.4	2.2±3.4	2.3±3.6	2.0±3.5
	SOM DASS-21 Dis	μ±σ	4.4±4.1	3.6±3.9	3.5±4.0	3.1±3.9
	Pain DR	Yes/No	389/210	NM	NM	NM
	Headache	B/S/No	70/281/247			
	PCI: active coping	μ±σ	24.6±6.2	23.6±6.7	NM	NM
	PCI: passive coping	μ±σ	35.8±9.0	33.6±9.4	NM	NM
	IPQ-k duration	μ±σ	4.1±2.7	NM	NM	NM
	IPQ-k treatment	μ±σ	7.8±1.9	NM	NM	NM
	IPQ-k concerns	μ±σ	4.0±2.6	3.7±2.5	2.9±2.5	2.6±2.5
	IPQ-k identity	μ±σ	6.1±2.4	2.9±2.5	6.2±2.4	6.3±2.5

-, not applicable; μ, average; σ, SD; B, still and before neck pain; BMI, body mass index; DASS, Depression Anxiety Stress Scale; Dep, depression; Dis, distress; F, female; IPQ-k, Illness Perception Questionnaire–kort (short form); M, male; NM, not measured; Pain DR, pain in different regions; PCI, Pain Coping Inventory; PCS, Pain Catastrophising Scale; PDI, Pain Disability Index; PSEQ-2, Pain Self-Efficacy Questionnaire 2-items; PVAQ, Pain Vigilance and Awareness Questionnaire; S, since neck pain; SOM, summed outcome of all items; TSK, Tampa Scale for Kinesiofobia;

### Longitudinal change in pain intensity

**Figure 1:**
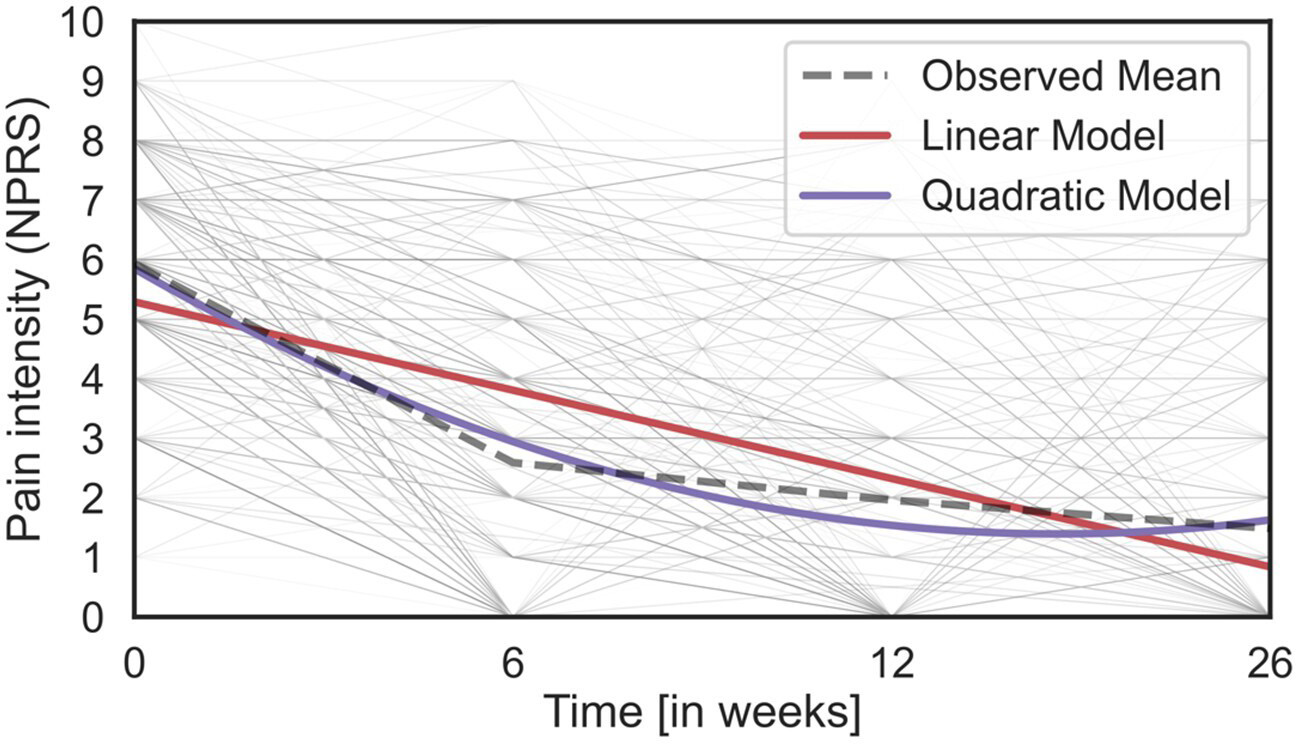
This figure displays the observed mean pain intensity (black dashed line), as well as the estimated trajectories from the linear model (red solid line) and quadratic model (blue solid line). Individual participant trajectories are shown in light grey to illustrate variability across subjects. NPRS, Numeric Pain Rating Scale.

First, the linear growth model was created to estimate the average rate of change in pain over time (table 2). Next, a quadratic term was added to the linear growth model to evaluate whether this term improved model fit. The LLRT indicated that the model with both a linear and quadratic term significantly improved the estimations (LR statistic=413, df=1, p<0.01). In the quadratic model, pain intensity showed a steep initial decline (Time=–0524, p<0.01), which further decelerated over time as indicated by the positive quadratic term (Time²=0.014, p<0.01). Random effect variance indicated substantial between-subject variability in initial pain levels. The model with both a linear and quadratic term was used for further analyses (figure 1).

**Table 2:** Growth model of the rate of change of pain over time

		Coefficient	SE	(95% CI)	Z	P value
Linear term	Intercept	5.288	0.091	(5.109 to 5.467)	57.882	<0.01
	Time	−1.483	0.044	(−1.569 to −1.397)	33.833	<0.01
	Random effect	1.684	0.102			
Linear term and quadratic term	Intercept	5.845	0.094	(5.661 to 6.030)	62.105	<0.01
	Time	−3.652	0.139	(−3.925 to −3.380)	−26.263	<0.01
	Time^2^	0.749	0.046	(0.659 to 0.839)	16.303	<0.01
	Random effect	1.858	0.110			

### Factors influencing pain trajectories

Modifiable clinical indicators were subsequently added as effect modifiers to the baseline growth-curve model (3.2), which included both a linear and quadratic term. Of the N 143 clinical indicators assessed, 24 showed a significant interaction with time and 13 with quadratic time, indicating an effect on the pain trajectory (table 3). Clinical indicators significantly associated with the linear coefficient reflected an increase or decrease in the rate of change of pain over time, that is, how quickly patients improved or deteriorated. The clinical indicators associated with the quadratic coefficient affected the shape of the trajectory, indicating whether improvement accelerated, slowed down, or began to plateau earlier or later. The results for all variables are provided in online supplemental appendix 3.

**Table 3:** Overview of significant effect modifiers

	Variable	Interaction effect (time)	Interaction effect (time2)	
	Coefficient (SE) (95% CI)	P value (95% CI)	P value (95% CI)	R^2^
Stress*	0.20 (0.03) (0.13 to 0.26)	**0.02**	0.11	0.49
Pain in different body regions	0.32 (0.18) (−0.02 to 0.67)	**<0.01**	**<0.01**	0.48
Headache	0.39 (0.05) (0.30 to 0.49)	**<0.01**	0.06	0.41
Feeling rested*	0.03 (0.18) (−0.32 to 0.37)	**<0.01**	**<0.01**	0.47
Pain disability (PDI)	0.06 (0.01) (0.05 to 0.07)	**<0.01**	**<0.01**	0.47
Coping (PCI Q:4)	0.24 (0.12) (−0.00 to 0.48)	**0.01**	**0.02**	0.45
Coping (PCI Q:5)*	0.51 (0.12) (0.28 to 0.74)	**0.02**	0.1	0.45
Coping (PCI Q:6)*	0.36 (0.11) (0.14 to 0.58)	**0.02**	**0.04**	0.45
Coping (PCI Q:8)	0.18 (0.12) (−0.05 to 0.41)	**<0.01**	**0.01**	0.45
Coping (PCI Q:13)*	0.14 (0.11) (−0.08 to 0.36)	**0.01**	0.07	0.46
Coping (PCI Q:26)*	0.38 (0.09) (0.21 to 0.55)	**<0.01**	**<0.01**	0.46
Coping (PCI Q:28)*	0.46 (0.10) (0.26 to 0.66)	**0.02**	**<0.01**	0.46
Reducing demands (PCI Subscale)	0.13 (0.05) (0.04 to 0.22)	**0.02**	**0.03**	0.45
Resting (PCI Subscale)	0.14 (0.04) (0.07 to 0.21)	**<0.01**	**<0.01**	0.46
PCS1_T0	0.38 (0.11) (0.16 to 0.60)	**0.03**	0.19	0.47
Duration beliefs (B-IPQ/IPQ-K Q:2)*	0.12 (0.03) (0.06 to 0.19)	**<0.01**	**<0.01**	0.49
Treatment beliefs (B-IPQ/IPQ-K Q:4)	0.09 (0.05) (−0.01 to 0.19)	**0.02**	0.16	0.46
Concerns (B-IPQ/IPQ-K Q:6)*	0.06 (0.06) (−0.06 to 0.18)	**0.05**	0.24	0.45
FT_PT_Rel_T0	0.20 (0.03) (0.13 to 0.26)	**0.02**	0.16	0.49
Tampa5_T0	−0.05 (0.17) (−0.38 to 0.28)	**0.03**	0.13	0.45
Tampa10_T0	0.07 (0.13) (−0.18 to 0.32)	**0.05**	0.37	0.46
Kinesofobia (TSK-11 Q:9)*	−0.10 (0.12) (−0.33 to 0.14)	**0.02**	0.08	0.45
SOMPSEQ_T0	−0.06 (0.03) (−0.13 to −0.00)	**<0.01**	**<0.01**	0.47
Attention to pain (PVAQ Q:7)*	0.36 (0.06) (0.24 to 0.47)	**0.04**	**0.02**	0.47

Only modifiable clinical indicators were included in this overview; the results of all variables are reported in online supplemental appendix 3. The clinical indicators marked with a were measured multiple times. Significant interaction effects were marked in bold.

* Variables measured at four time points (T0–T3).

B-IPQ, Brief Illness Perception Questionnaire; FT_PT_Rel, physiotherapist–patient relation; IPQ-K, Illness Perception Questionnaire-Kort (short version); PCI, Pain Coping Inventory; PCS, Pain Catastrophising Scale; PDI, Pain Disability Index; PSEQ-2, Pain Self-efficacy Questionnaire 2-item short form; PVAQ, Pain Vigilance Awareness Questionnaire; R2, marginal R2; TSK-11, Tampa Scale of Kinesiofobia 11-item version.

### Collinearity among clinical indicators

**Figure 2:**
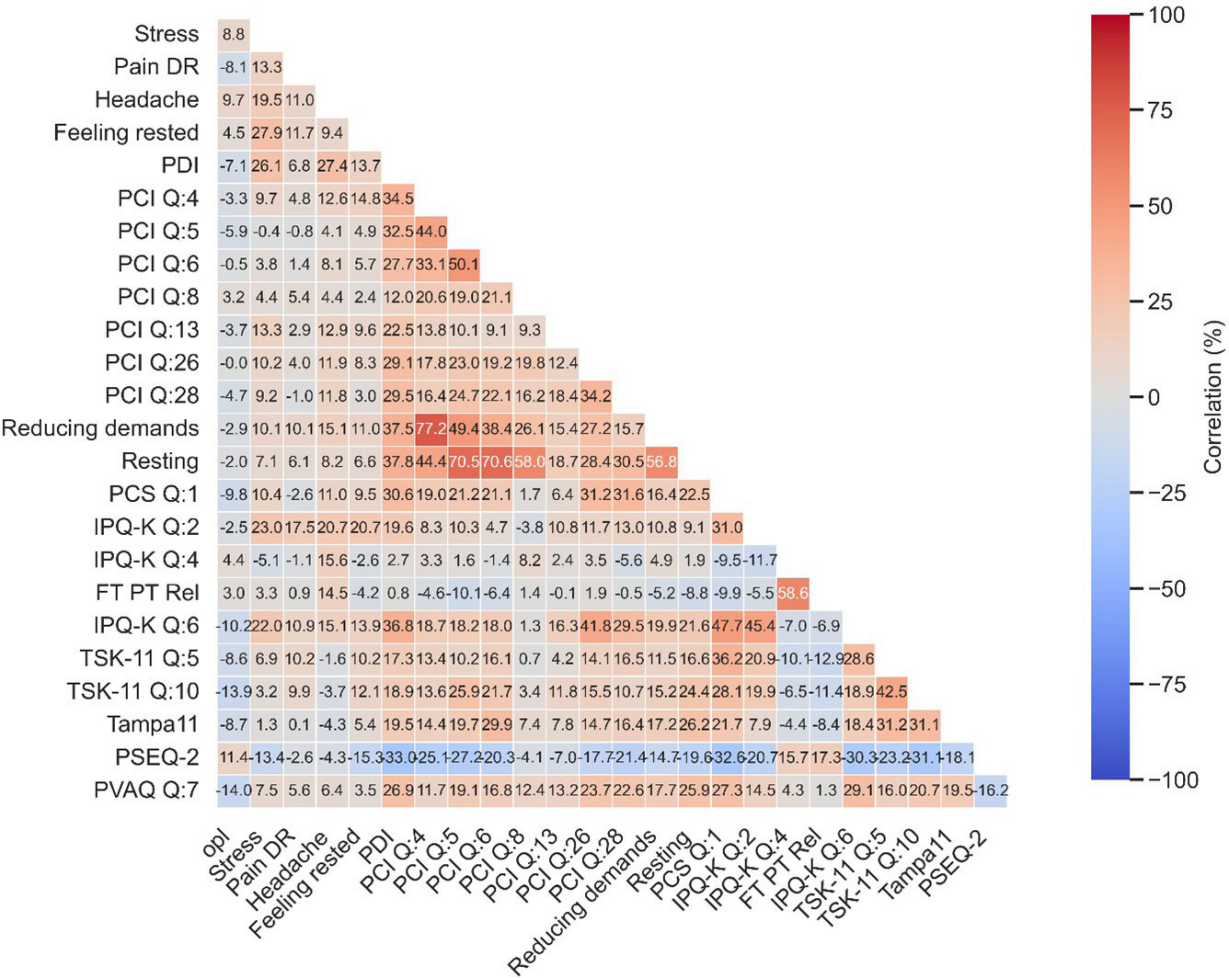
Pearson’s correlation coefficient of modifiable clinical indicators significantly associated with pain recovery. DR, different region; FT PT Rel, physiotherapist–patient relation; IPQ-K, Illness Perception Questionnaire-Kort (short version); PCI, Pain Coping Inventory; PCS, Pain Catastrophising Scale; PDI, Pain Disability Index; PSEQ, Pain Self-efficacy Questionnaire 2-item; PVAQ, Pain Vigilance Awareness Questionnaire; TSK-11, Tampa Scale for Kinesiofobia.

The correlation coefficient of modifiable clinical indicators associated with pain recovery was evaluated with the Pearson’s correlation coefficient at baseline (figure 2). Three variables showed high correlations (|r|>70%). Only reducing demands (PCI subscale) showed a high correlation with item four of the PCI (PCI4: ‘If I am in pain, I continue my activities, but less precise’) (r=77.2). Similarly, resting (PCI subscale) was highly correlated with item 5 (PCI5: ‘When I am in pain, I confine myself to simple activities’) and item 6 of the PCI (PCI6: ‘When I am in pain, I take care that I don’t have to exert myself physically’) (r=70.6). To minimise patient burden and redundancy, the corresponding subscale scores were excluded from the final compass.

### Visualisation of the factors for clinical use

**Figure 3:**
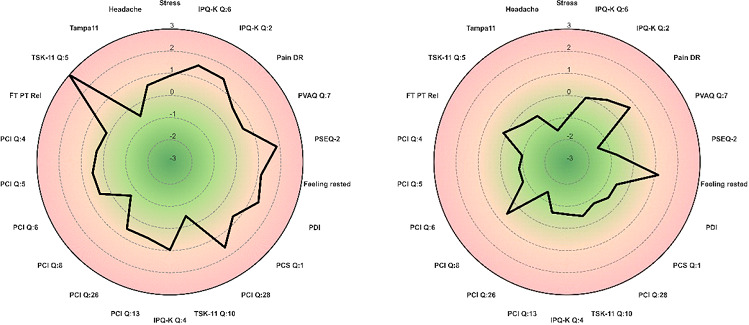
Two radar charts illustrating the standardised scores (Z-scores) of an individual patient across the 22 modifiable factors. The shaded area depicts the patient’s profile in relation to the group mean (Z=0, indicated by the dashed circle). Positive values indicate outcomes above the group average, whereas negative values indicate outcomes below average. The background shading indicates the direction and magnitude of deviation from the group mean (green=below average; red=above average). The clinical indicators in the graph are arranged clockwise according to their explained variance (marginal R2), starting with the IPQ-K Q:6. The IPQ-K: Q:4 and the PSEQ-2 were reverse-scored for the radar chart so that higher values consistently indicate a less favourable profile. The right chart (patient 158) shows a relatively confined pattern, with higher scores on feeling rested and on selected individual items related to pain self-efficacy and illness perceptions (IPQ-K Q:2 and Q:4), but notably lower scores on specific questions addressing perceived control (PCI items), activity-related fear (TSK-11 Q:5). This patient reported a baseline pain intensity (NPRS) of 4, which decreased to 0 at 6 months. In contrast, the left chart (patient 514) demonstrates a broader deviation from the mean, with elevated scores on individual questions reflecting stress, illness perceptions (IPQ-K Q:2 and Q:6), pain-related disability (PDI), pain in different body regions (Pain DR). This patient reported a baseline NPRS of 7 and a score of 6 at 6 months. This visualisation highlights the heterogeneity of patient profiles and allows rapid identification of individual strengths and limitations. FT PT Rel, physiotherapist–patient relation; IPQ-K, Illness Perception Questionnaire-Kort (short version); NPRS, Numeric Pain Rating Scale; PCI, Pain Coping Inventory; PCS, Pain Catastrophising Scale; PDI, Pain Disability Index; PSEQ, Pain Self-efficacy Questionnaire 2-item; PVAQ, Pain Vigilance Awareness Questionnaire; TSK-11, Tampa Scale for Kinesiofobia.

Table 4 provides an overview of the final clinical indicators included in the clinical compass. The 22 remaining clinical indicators were incorporated into a radar chart to facilitate visualisation of patient-specific results. Figure 3 illustrates the resulting radar chart, showing two examples of patient data at baseline.

**Table 4:** Overview of final clinical indicators included in the clinical compass

Clinical indicator	English version	Outcome	Dutch version	Outcome	Indicator of factor
Stress	Do you feel stressed?	Scale from 0 (not at all stressed) to 10 (very stressed)	Voelt u zich gestrest?	Schaal van 0 (helemaal niet gestrest) tot 10 (zeer gestrest)	Stress
WSP	Do you experience pain in other parts of the body?	Yes/No	Pijn in andere plekken van het lichaam	Ja/Nee	Pain in different body regions
Headache	Could you indicate how much headache you experience on a scale from 0 to 10?	Scale from 0 (no headache at all) to 10 (worst imaginable headache)	Kunt u aangeven hoeveel hoofdpijn u ervaart op een schaal van 0–10?	Schaal van 0 (helemaal geen hoofdpijn) tot 10 (meest denkbare hoofdpijn)	Headache
Feeling rested	I do not wake up feeling rested in the morning.	Yes: I wake up feeling rested in the morning. No: I do not wake up feeling rested in the morning.	Ik word ’s morgens niet uitgerust wakker.	Ja: ik word ’s morgens uitgerust wakker, Nee: ik word ’s morgens niet uitgerust wakker	Sleep quality
PDI	For each of the following areas of your life, please rate the degree to which your pain currently interferes with your usual activities. Family and home responsibilities Recreation Social activity Occupation Sexual behaviour Self-care Life-support activity	Scale from 0 (no disability) to 10 (total disability)	Wilt u per onderdeel aangeven in welke mate u door uw pijn wordt belemmerd? Familiaire en huishoudelijke verantwoordelijkheden Recreatie Sociale activiteiten Beroep Seksuele activiteiten Zelfverzorging Basale levensbehoeftes. 0 (geen beperkingen) tot 10 (totaal beperkt).	Schaal van 0 (geen beperkingen) tot 10 (volledig beperkt)	Pain disability
PCI Q:4	When I am in pain, I continue my activities, but less precise.	Hardly ever sometimes often very often	Wanneer ik pijn heb, ga ik door met mijn bezigheden, maar minder nauwgezet.	Zelden of nooit soms vaak zeer vaak	Coping: Reducing demands
PCI Q:5	When I am in pain, I confine myself to simple activities.	Hardly ever sometimes often very often	Wanneer ik pijn heb, beperk ik me tot eenvoudige bezigheden.	Zelden of nooit soms vaak zeer vaak	Coping: Resting
PCI Q:6	When I am in pain, I take care that I don't have to exert myself physically.	Hardly ever sometimes often very often	Wanneer ik pijn heb, zorg ik dat ik me niet lichamelijk hoef in te spannen.	Zelden of nooit soms vaak zeer vaak	Coping: Resting
PCI Q:8	When I am in pain, I take on a comfortable bodily posture.	Hardly ever sometimes often very often	Wanneer ik pijn heb, neem ik een prettige lichaamshouding aan.	Zelden of nooit soms vaak zeer vaak	Coping: Resting
PCI Q:13	When I am in pain, I take care that I am not bothered by the light (eg, by putting on sunglasses, closing the curtains).	Hardly ever sometimes often very often	Wanneer ik pijn heb, zorg ik ervoor dat ik niet gehinderd word door het licht (bv. door een zonnebril op te zetten, de gordijnen dicht te doen).	Zelden of nooit soms vaak zeer vaak	Coping: retreating
PCI Q:26	When I am in pain, I wonder about the cause of the pain.	Hardly ever sometimes often very often	Wanneer ik pijn heb, vraag ik me af wat de oorzaak van de pijn is.	Zelden of nooit soms vaak zeer vaak	Coping: Worrying
PCI Q:28	When I am in pain, I think of moments when I was not in pain.	Hardly ever sometimes often very often	Wanneer ik pijn heb, denk ik aan momenten waarop ik geen pijn had.	Zelden of nooit soms vaak zeer vaak	Coping: Worrying
PCS Q:1	When I am in pain, I worry all the time about whether the pain will end.	Not at all to a slight degree to a moderate degree to a great degree all the time	Als ik lichamelijke klachten heb, vraag ik mij voortdurend af of de klachten wel zullen ophouden.	helemaal niet van toepassingeen beetje van toepassingenigszins van toepassingbehoorlijk van toepassingheel erg van toepassing	Catastrophising
B-IPQ/IPQ-K Q:2	How long do you think your illness will continue?	Scale from 0 (a very short time) to 10 (forever)	Hoe lang denkt u dat uw nekpijn zal duren?	Schaal van 0 (een zeer korte tijd) tot 10 (mijn hele leven)	Illness Perception: Duration
B-IPQ/IPQ-K Q:4	How much do you think your treatment can help your illness?	Scale from 0 (not at all) to 10 (extremely helpful)	Hoeveel denkt u dat uw behandeling kan helpen bij uw nekpijn?	Schaal van 0 (helemaal niet) tot 10 (zeer veel)	Illness Perception: Treatment
B-IPQ/IPQ-K Q:6	How concerned are you about your illness?	Scale from 0 (not at all concerned) to 10 (extremely concerned)	Hoe bezorgd bent u over uw ziekte?	Schaal van 0 (helemaal niet bezorgd) tot 10 (zeer bezorgd)	Illness Perception: Concerns
TSK-11 Q:5	People aren’t taking my medical condition seriously enough.	Strongly disagree disagree agree strongly agree)	Mijn gezondheidstoestand wordt door anderen niet serieus genoeg genomen.	in hoge mate mee oneens enigszins mee oneens enigszins mee eens in hoge mate mee eens	Kinesiofobia
TSK-11 Q:10	Simply being careful that I do not make any unnecessary movements is the safest thing I can do to prevent my pain from worsening.	Strongly disagree disagree agree strongly agree)	De veiligste manier om te voorkomen dat mijn pijn erger wordt is gewoon oppassen dat ik geen onnodige bewegingen maak.	in hoge mate mee oneens enigszins mee oneens enigszins mee eens in hoge mate mee eens	Kinesiofobia
TSK-11 Q:9	I am afraid that I might injure myself accidentally.	Strongly disagree disagree agree strongly agree)	Mijn pijn zegt me wanneer ik moet stoppen met lichaamsoefeningen doen om geen letsel op te lopen	in hoge mate mee oneens enigszins mee oneens enigszins mee eens in hoge mate mee eens	Kinesiofobia
PSEQ-2	I can still accomplish most of my goals in life, despite the pain. I can live a normal lifestyle, despite the pain.	Scale from 0 (not at all confident) to 6 (completely confident) Sum score of both questios	Ik kan nog steeds de meeste van mijn doelen in mijn leven halen, ondanks de pijn Ik kan een normaal leven leiden, ondanks de pijn.	Schaal van 0 (geheel geen vertrouwen) tot 6 (volledig vertrouwen) Somscore van beide items	Pain Self-efficacy
PVAQ Q:7	I notice pain even if I am busy with another activity.	Scale from 0 (never) to 5 (always)	Als ik pijn heb, ben ik me daar zeer bewust van, zelfs wanneer ik druk bezig ben met andere activiteiten	Schaal van 0 (nooit) tot 5 (steeds)	Pain and Vigilance Awareness: Attention to pain
Therapeutic relation	How much trust do you have in your healthcare provider/physiotherapist?	Scale from 0 (no trust at all) to 10 (very much confidence)	Hoeveel vertrouwen heeft u in uw zorgverlener/fysiotherapeut?	Schaal van 0 (helemaal geen vertrouwen) tot 10 (heel veel vertrouwen)	Therapeutic relation

B-IPQ, Brief Illness Perception Questionnaire; IPQ-K, Illness Perception Questionnaire-Kort (short version); PCI, Pain Coping Inventory; PCS, Pain Catastrophizing Scale; PDI, Pain Disability Index; PSEQ-2, Pain Self-efficacy Questionnaire 2-item short form; PVAQ, Pain Vigilance Awareness Questionnaire; Q, question; TSK-11, Tampa Scale of Kinesiofobia 11-item version; WSP, widespread pain.

## Discussion

Biological, psychological and behavioural clinical indicators were identified as being associated with the rate of change in pain intensity over time in patients with NSNP. Building on these findings, we developed a 22-item clinical compass, comprising single-item indicators and two brief multi-item measures, to provide a concise patient-specific profile. The clinical compass visualises this profile to support individualised clinical reasoning and to help structure further assessment of potentially relevant domains in primary care physiotherapy.

### Comparison with previous research

The present study employed a longitudinal, data-driven approach, incorporating repeated pain measurements, which allowed for a practice-relevant understanding of how pain develops. Previous studies in musculoskeletal pain have often relied on dichotomous outcome measures, sometimes assessed at a single time point.^17 41–46^ Such approaches provide only a limited understanding of the dynamic nature of pain, as they fail to capture how symptoms fluctuate and evolve over time. From a patient perspective, pain commonly varies from day to day, and single-time assessments may therefore provide a distorted view of the recovery process. Our study perspective more accurately reflects the clinical reality of physiotherapy, where recognising and interpreting patterns of change is essential for delivering personalised care. In comparison with the widely used STarT MSK, the clinical compass reflects a different conceptual approach. Whereas the STarT MSK was developed as a brief stratification tool to support risk-based care pathways.^47^ The clinical compass was developed as a profiling tool intended to visualise potentially modifiable clinical indicators and support individualised clinical reasoning. Although both approaches address comparable conceptual domains, such as duration beliefs, multisite pain, pain interference, self-efficacy and kinesiophobia, they differ in both purpose and structure. The clinical compass does not aim to classify patients into broad risk categories, but to provide a patient-specific overview of clinically relevant indicators that may guide further assessment and inform personalised care.

### Measurement considerations

Several items in the clinical compass capture relevant aspects of the psychosocial and behavioural domains, but do not fully represent the underlying constructs. In these domains, coping subdomains, reducing demands (one item), resting (three items), worrying (two items), retreating (one item), as well as catastrophising, kinesiophobia and hypervigilance, are represented by selected items that capture elements of these constructs.^30–32 48^ Self-efficacy is assessed using a short, validated two-item instrument,^29^ and selected illness perceptions are captured using individual items from a short, validated form.^28^ In contrast, stress, pain in other body regions, headache and feeling rested are measured with single questions that are not validated as stand-alone measures. Pain disability is assessed using a complete, validated questionnaire.^25^

We analysed both the total scores of the questionnaires and their constituent items. Single-item clinical indicators showed stronger associations with within-person changes in pain over time than their corresponding total scores, supporting their inclusion in the clinical compass. At the same time, several items have not been validated as independent measures; therefore, the clinical compass should be regarded as a clinical reasoning-support instrument rather than a replacement for full questionnaires that measure complete constructs. Moreover, single items generally demonstrate larger measurement error than combined scale scores, which may attenuate the fixed-effect coefficients in the mixed models, meaning the true associations between these indicators and pain intensity could be somewhat larger than reported.

Relatedly, outcome selection was constrained by the cohort’s repeated-measures structure. Comparable longitudinal data were not available for other clinically relevant outcomes, such as disability or global recovery, and these outcomes could therefore not be examined using similar trajectory analyses in the present study. Future longitudinal research should examine whether these outcomes provide complementary insights into recovery trajectories.

### Methodological limitations

The first limitation concerns the use of the NPRS as an outcome, which is known to show considerable within-individual variation.^49 50^ In this study, the NPRS prompt did not specify a recall period or whether to report current versus average pain; responses therefore reflect momentary ratings with uncertain interpretation, increasing measurement error and potentially attenuating associations.

Second, because this study used a data-driven approach with a large candidate set, the risk of type I error cannot be entirely excluded.^51^ We mitigated this risk by restricting analyses to prespecified domains (ie, a priori defined, clinically plausible domains with established relevance to NSNP) and interpreting the results in light of established authors’ clinical expertise. Reassuringly, the significant clinical indicators largely align with prior literature.^52–56^ Nonetheless, replication in an independent dataset is warranted. By contrast, theoretically relevant factors that did not emerge, most notably catastrophising, raise the possibility of type II error and/or restricted variability in our sample, both at the total-score level and at the single-item level.

Finally, the current version of the clinical compass presents results in a radar chart using standardised scores (z-scores) to show how an individual patient deviates from the cohort mean. At this stage, these deviations are intended to support structured clinical reasoning rather than to function as prescriptive thresholds. The visualised profile may help identify domains for further assessment, but does not yet indicate whether observed differences are clinically meaningful. Future research should therefore investigate how the interpretability and utility of the clinical compass can be strengthened, including the potential development of clinically relevant reference values.

### Strengths and limitations

A key strength of this study is its direct clinical applicability: we developed a clinical compass to help physiotherapists identify relevant biopsychosocial clinical indicators early in the treatment process. By making such clinical indicators explicit, the clinical compass may support more informed and structured individualised clinical reasoning. However, the clinical compass should be understood as a profiling tool rather than as evidence that changing the identified indicators will necessarily alter pain trajectories. The present analyses were associative in nature and did not evaluate mediation, temporal causal ordering, or treatment effects. Therefore, the compass is intended to help clinicians identify potentially relevant domains for further assessment, rather than to imply that modifying an identified indicator will automatically improve outcomes.

A further consideration is that the analyses focused on indicators that are potentially modifiable and may change over time.^8 24^ Not all indicators were collected at every time point; however, a subset was assessed repeatedly (T1–T3), and these repeated measurements were included in the analyses to capture within-person change. Online supplemental appendix 4 provides a descriptive overview of temporal variation in these variables and their relation to the mean change in pain. Nonetheless, we did not estimate causal effects of indicator changes on pain trajectories. In addition, participants received usual physiotherapy care in routine primary care practice, and treatment was not standardised across physiotherapists or practices. Such variation may have influenced pain trajectories independently of the indicators included in the clinical compass and was not accounted for in the present analyses.

Future research should therefore examine the extent to which these factors fluctuate during recovery and whether such changes track with subsequent shifts in pain trajectories, including disentangling natural recovery from treatment effects.

### Implications for research

Future research should focus first on the further development and validation of the clinical compass. This includes (1) examining the content validity of the selected clinical indicators (eg, through cognitive interviewing with patients), (2) establishing the test–retest reliability and measurement error of the single items, (3) evaluating their responsiveness of the single questions, (4) and investigating whether targeting and modifying these items in clinical practice is associated with subsequent changes in pain trajectories. Finally, future studies with larger samples or more measurement timepoints should consider extending the current modelling approach by incorporating random slopes, allowing individual trajectories to vary across participants, or by exploring non-LMMs to better capture the complexity of pain trajectories over time.

In addition, studies are needed to assess the usability, interpretability, and clinical utility of the clinical compass as a tool to support structured assessment and individualised clinical reasoning. Finally, practice-based research, such as pragmatic prospective studies or single-case experimental designs, should examine whether use of the clinical compass in assessment and decision-making is associated with differences in care processes, clinical reasoning, and pain trajectories over time across independent samples and settings.

### Implications for practice

Despite broad recognition of the value of early psychosocial assessment in musculoskeletal care, routine use of psychosocial screening tools or complete questionnaires by physiotherapists remains limited.^57^ Only a minority of physiotherapists (37%) routinely use screening tools, and 22% use risk stratification tools.^57^ Reported barriers include inadequate training, uncertainty in interpreting results, limited time and resources, and concerns about possible stigmatisation of patients with psychosocial risk factors.^57^

In this context, the clinical compass may offer a brief and practice-oriented way of structuring patient-specific assessment. Its potential value in practice will depend on further evaluation of usability, interpretability, and integration into routine care. Future implementation efforts would likely require targeted training to help therapists interpret the clinical compass appropriately, integrate it into routine assessment, and strengthen therapists’ skills to address potential modifiable factors,^58 59^ and careful consideration of how the compass could be incorporated into existing clinical workflows.

## Conclusions

This study identified several biological, psychological and behavioural clinical indicators associated with change in pain intensity over time in patients with NSNP. The indicators were integrated into a 22-item prototype clinical compass to visualise patient-specific profiles and support prognostic reasoning in primary care. The findings are exploratory, reflect associations observed in a Dutch primary care physiotherapy context, and require replication, external validation and evaluation of usability and clinical impact before clinical implementation.

## Supplementary material

10.1136/bmjopen-2025-115274 online supplemental appendix 1

10.1136/bmjopen-2025-115274 online supplemental appendix 2

10.1136/bmjopen-2025-115274 online supplemental appendix 3

10.1136/bmjopen-2025-115274 online supplemental appendix 4

## Data Availability

Data are available in a public, open access repository.
