# Supplementary material for: Clinical indicators associated with pain trajectories in non-specific neck pain: a secondary analysis of a multicentre prospective observational cohort to develop a clinical compass for personalised physiotherapy in primary care
Source: BMJ Open. 2026 Aug 3;16(8):e115274. doi: 10.1136/bmjopen-2025-115274 (PMC13435912; doi:10.1136/bmjopen-2025-115274)
Supplement: Supplementary file 1 [file bmjopen-16-8-s001.pdf]

Appendix 1

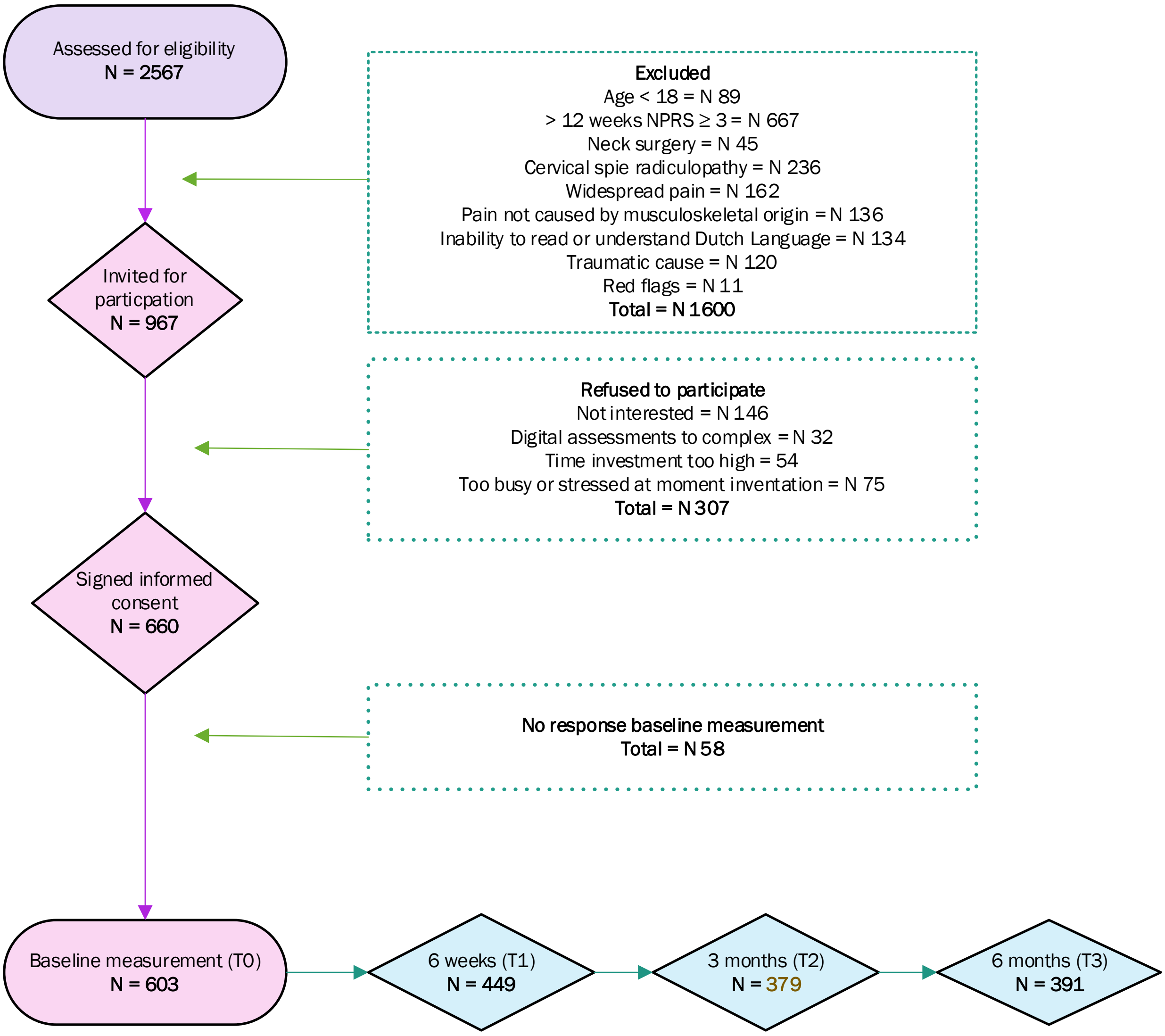

Flow-chart study, reporting numbers at each stage (potentially eligible, assessed for eligibility, confirmed eligible, included, completed follow-up.

Abbreviations: NPRS, Numeric Pain Rating Scale; N, number; T, time-point.
