# Supplementary material for: Clinical indicators associated with pain trajectories in non-specific neck pain: a secondary analysis of a multicentre prospective observational cohort to develop a clinical compass for personalised physiotherapy in primary care
Source: BMJ Open. 2026 Aug 3;16(8):e115274. doi: 10.1136/bmjopen-2025-115274 (PMC13435912; doi:10.1136/bmjopen-2025-115274)
Supplement: Supplementary file 2 [file bmjopen-16-8-s002.pdf]

Appendix 2

Availability of participant characteristics and clinical indicators across time points (n = 603)

		0 weeks	6 weeks	12 weeks	26 weeks
Patient Characteristics	Age	602 [100%]	-	-	-
	Sex	603 [100%]	-	-	-
	Duration	599 [99%]	-	-	-
	Recurrent	602 [100%]	-	-	-
	Education	587 [97%]	-	-	-
	BMI	591 [98%]	-	-	-
Patient reported outcome measures	Pain intensity	603 [100%]	277 [74%]	175 [64%]	135 [65%]
	SOM PDI	602 [100%]	267 [44%]	NM	NM
	SOM PCS	600 (100%]	432 [71%]	371 [61.5%]	330 [54.7%]
	SOM TSK	600 [100%]	429 [71.4%]	NM	NM
	SOM PSEQ-	601 [100%]	407 [67%]	169 [28%]	119 [19.7%]
	SOM PVAQ	600 [100%]	425 [70%]	364 [60%]	326 [54%]
	SOM DASS-21 Dep	600 [100%]	424 [70%]	362 [60%]	324 [54%]
	SOM DASS-21 Dis	600 [100%]	425 [70%]	362 [60%]	324 [54%]
	Pain DR	599 [99%]	NM	NM	NM
	Headache	598 [99%]			
	PCI: Active Coping	600 [100%]	419 [69%]	NM	NM
	PCI: Passive coping	600 [100%]	419 [69%]	NM	NM
	IPQ-k duration	593 [98%]	NM	NM	NM
	IPQ-k treatment	591 [98%]	NM	NM	NM
	IPQ-k concerns	595 [99%]	432 [72%]	369 [61%]	329 [55%]
	IPQ-k identity	595 [99%]	430 [71%]	368 [61%]	327 [54%]

Abbreviations: μ = Average; σ = Standard deviation; - = Not Applicable; M = Male; F = Female; B = still and before neck pain; S = since neck pain; SOM = summed outcome of all items; PDI = Pain Disability Index; NM = not measured; PCS = Pain Catastrophizing Scale; TSK = Tampa Scale for Kinesiofobia; PSEQ-2 = Pain Self-Efficacy Questionnaire 2-items; PVAQ = Pain Vigilance and Awareness Questionnaire; DASS = Depression Anxiety Stress Scale; Dep = Depression, Dis = Distress; Pain DR = pain in different regions; PCI = Pain Coping Inventory; IPQ-k: Illness Perception Questionnaire – kort (short form)
