# Supplementary material for: Clinical indicators associated with pain trajectories in non-specific neck pain: a secondary analysis of a multicentre prospective observational cohort to develop a clinical compass for personalised physiotherapy in primary care
Source: BMJ Open. 2026 Aug 3;16(8):e115274. doi: 10.1136/bmjopen-2025-115274 (PMC13435912; doi:10.1136/bmjopen-2025-115274)
Supplement: Supplementary file 3 [file bmjopen-16-8-s003.pdf]

## Appendix 3

Overview of all effect modifiers.

| | **Variable** C(SE) | **Linear term (time)** C(SE) | **Interaction Effect (time)** C(SE) | **Interaction Effect (time)** P [95%] | **Quadratic term ($time^2$)** C(SE) | **Interaction Effect ($time^2$)** C(SE) | **Interaction Effect ($time^2$)** P [95%] |
|---|---|---|---|---|---|---|---|
| Sex | .25 (.20) | -4.41 (.51) | .45 (.29) | .12 [-.12, 1.03] | 1.01 (.17) | -.16 (.10) | .11 [-.35, .04] |
| Age | .01 (.01) | -3.77 (.42) | .00 (.01) | .8 [-.02, .02] | .78 (.14) | -.00 (.00) | .86 [-.01, .01] |
| Length | -.01 (.01) | .32 (2.52) | -.02 (.01) | .11 [-.05, .01] | -.45 (.83) | .01 (.00) | .15 [-.00, .02] |
| Weight | .00 (.01) | -3.42 (.71) | -.00 (.01) | .7 [-.02, .01] | .71 (.23) | .00 (.00) | .83 [-.01, .01] |
| BMI | .02 (.02) | -4.01 (.84) | .01 (.03) | .69 [-.05, .08] | .91 (.28) | -.01 (.01) | .58 [-.03, .02] |
| Smoking | .57 (.29) | -3.85 (.52) | .19 (.45) | .68 [-.70, 1.07] | .79 (.17) | -.04 (.15) | .79 [-.33, .25] |
| Smoking per day | .03 (.05) | -4.17 (.91) | .07 (.07) | .37 [-.08, .21] | 1.04 (.31) | -.03 (.03) | .19 [-.08, .02] |
| Alcohol | .07 (.23) | -3.35 (.62) | -.18 (.34) | .6 [-.84, .48] | .76 (.20) | -.00 (.11) | .98 [-.22, .21] |
| Degree of alcohol use | -.09 (.12) | -3.85 (.48) | .06 (.17) | .72 [-.28, .40] | .85 (.16) | -.04 (.06) | .52 [-.15, .08] |
| Education level | -.03 (.06) | -2.86 (.50) | -.13 (.08) | .11 [-.29, .03] | .60 (.17) | .02 (.03) | .38 [-.03, .08] |
| Work | .09 (.25) | -3.23 (.47) | -.34 (.38) | .36 [-1.08, .40] | .55 (.15) | .16 (.12) | .18 [-.08, .41] |
| Happiness at work | -.12 (.13) | -4.34 (.50) | .34 (.22) | .11 [-.08, .77] | .90 (.17) | -.08 (.07) | .28 [-.22, .06] |
| Happiness at work(dich) | -.26 (.24) | -3.97 (.48) | .28 (.36) | .43 [-.43, .99] | .83 (.16) | -.08 (.12) | .49 [-.32, .15] |
| Job satisfaction | -.16 (.15) | -3.76 (.52) | .09 (.24) | .69 [-.37, .56] | .72 (.17) | -.00 (.08) | .98 [-.15, .15] |
| Job satisfaction(dich) | -.29 (.25) | -3.81 (.52) | .22 (.41) | .59 [-.59, 1.03] | .73 (.17) | -.02 (.13) | .91 [-.28, .25] |
| Self-modify posture | -.13 (.12) | -4.35 (.44) | .37 (.21) | .07 [-.03, .78] | .90 (.15) | -.08 (.07) | .22 [-.22, .05] |
| Self-modify posture(dich) | -.18 (.23) | -4.05 (.51) | .36 (.40) | .37 [-.42, 1.13] | .76 (.17) | -.02 (.13) | .87 [-.28, .24] |
| Duration | -.01 (.03) | -4.22 (.25) | .12 (.05) | <.01 [.03, .21] | .85 (.08) | -.02 (.02) | .15 [-.05, .01] |
| Stress | .18 (.03) | -3.84 (.25) | .12 (.05) | .02 [.02, .23] | .81 (.08) | -.02 (.02) | .17 [-.06, .01] |
| Recurrence | .23 (.20) | -4.41 (.52) | .44 (.30) | .14 [-.14, 1.03] | .93 (.17) | -.11 (.10) | .28 [-.30, .09] |
| Recurrence (last episode) | -.00 (.00) | -3.37 (.22) | -.02 (.01) | .04 [-.03, -.00] | .67 (.07) | .01 (.00) | .04 [.00, .01] |
| Pain in different body regions | .24 (.18) | -5.48 (.48) | 1.25 (.30) | <.01 [.67, 1.83] | 1.18 (.15) | -.28 (.10) | <.01 [-.47, -.09] |
| Headache | .32 (.14) | -4.20 (.39) | .32 (.21) | .13 [-.10, .75] | .92 (.13) | -.10 (.07) | .16 [-.24, .04] |
| Headache Pain NPRS | .39 (.05) | -3.05 (.78) | .33 (.14) | .02 [.05, .61] | .62 (.27) | -.08 (.05) | .13 [-.18, .02] |
| Rest | .03 (.18) | -4.70 (.23) | 1.44 (.38) | <.01 [.69, 2.19] | 1.05 (.07) | -.37 (.12) | <.01 [-.61, -.12] |
| Hours awake | .27 (.08) | -4.17 (.25) | .00 (.18) | .99 [-.34, .35] | .89 (.08) | .02 (.06) | .75 [-.09, .13] |
| Self-reported sleep quality | .41 (.18) | -4.42 (.27) | .37 (.37) | .31 [-.35, 1.10] | .99 (.08) | -.11 (.12) | .35 [-.34, .12] |
| Falling asleep | .18 (.20) | -3.69 (.16) | .27 (.36) | .46 [-.44, .97] | .76 (.05) | -.02 (.12) | .84 [-.27, .22] |
| Sleep quality (dich) | .40 (.23) | -3.43 (.30) | -.27 (.34) | .42 [-.94, .39] | .70 (.10) | .06 (.11) | .58 [-.16, .28] |
| NNGB | .02 (.14) | -3.19 (.52) | -.18 (.21) | .38 [-.59, .23] | .59 (.17) | .06 (.07) | .35 [-.07, .20] |
| PDI Q: 1 | .31 (.03) | -2.14 (.24) | -.02 (.07) | .77 [-.16, .12] | .51 (.09) | .01 (.03) | .84 [-.05, .06] |
| PDI Q: 2 | .30 (.03) | -2.16 (.27) | .01 (.07) | .87 [-.13, .15] | .56 (.10) | -.02 (.03) | .53 [-.07, .03] |
| PDI Q: 3 | .29 (.03) | -2.40 (.22) | .10 (.08) | .2 [-.05, .25] | .62 (.08) | -.04 (.03) | .14 [-.10, .01] |
| PDI Q: 4 | .28 (.03) | -2.38 (.26) | .09 (.07) | .17 [-.04, .22] | .59 (.09) | -.03 (.03) | .26 [-.08, .02] |
| PDI Q: 5 | .27 (.03) | -2.28 (.21) | .02 (.07) | .78 [-.12, .16] | .56 (.08) | -.01 (.03) | .66 [-.06, .04] |
| PDI Q: 6 | .25 (.03) | -2.27 (.20) | .02 (.08) | .8 [-.13, .17] | .54 (.07) | -.01 (.03) | .79 [-.06, .05] |
| PDI Q: 7 | .24 (.03) | -2.36 (.21) | .10 (.08) | .18 [-.05, .25] | .58 (.08) | -.04 (.03) | .19 [-.09, .02] |
| Mean PDI | .40 (.04) | 17.36 (1095201.91) | -16.10 (nan) | nan [nan, nan] | -5.99 (365067.30) | 5.37 (nan) | nan [nan, nan] |
| Total Score PDI | .06 (.01) | -2.92 (.23) | -.04 (.01) | <.01 [-.06, -.02] | .58 (.08) | .01 (.00) | <.01 [.00, .02] |
| PCI Q: 1 | .60 (.13) | -3.39 (.39) | -.14 (.24) | .57 [-.60, .33] | .73 (.13) | .01 (.08) | .86 [-.14, .17] |
| PCI Q: 2 | .24 (.11) | -3.36 (.44) | -.13 (.19) | .48 [-.50, .24] | .71 (.14) | .03 (.06) | .69 [-.10, .15] |
| PCI Q: 3 | .33 (.10) | -3.38 (.40) | -.12 (.18) | .5 [-.48, .23] | .72 (.13) | .02 (.06) | .79 [-.10, .14] |
| PCI Q: 4 | .24 (.12) | -2.92 (.34) | -.41 (.18) | .02 [-.77, -.06] | .55 (.11) | .11 (.06) | .07 [-.01, .23] |
| PCI Q: 5 | .50 (.12) | -3.16 (.35) | -.31 (.20) | .12 [-.70, .08] | .69 (.12) | .05 (.07) | .48 [-.08, .17] |
| PCI Q: 6 | .37 (.11) | -2.97 (.35) | -.42 (.20) | .03 [-.82, -.03] | .57 (.12) | .12 (.07) | .07 [-.01, .25] |
| PCI Q: 7 | .27 (.10) | -3.40 (.38) | -.11 (.18) | .52 [-.46, .23] | .67 (.12) | .05 (.06) | .44 [-.07, .16] |
| PCI Q: 8 | .18 (.12) | -2.57 (.46) | -.42 (.17) | .02 [-.77, -.08] | .48 (.15) | .10 (.06) | .07 [-.01, .22] |
| PCI Q: 9 | .34 (.10) | -3.17 (.34) | -.22 (.14) | .12 [-.49, .06] | .62 (.11) | .06 (.05) | .23 [-.03, .15] |
| PCI Q: 10 | .24 (.11) | -3.20 (.33) | -.26 (.17) | .13 [-.59, .07] | .65 (.11) | .06 (.06) | .32 [-.05, .16] |
| PCI Q: 11 | .09 (.10) | -3.85 (.35) | .13 (.18) | .48 [-.23, .49] | .81 (.12) | -.03 (.06) | .6 [-.15, .09] |
| PCI Q: 12 | .07 (.11) | -3.98 (.33) | .22 (.19) | .25 [-.15, .59] | .83 (.11) | -.05 (.06) | .44 [-.17, .08] |
| PCI Q: 13 | .14 (.11) | -4.13 (.29) | .32 (.17) | .06 [-.01, .65] | .84 (.10) | -.06 (.06) | .29 [-.17, .05] |
| PCI Q: 14 | .10 (.10) | -3.76 (.29) | .07 (.15) | .66 [-.23, .36] | .74 (.10) | .01 (.05) | .9 [-.09, .10] |
| PCI Q: 15 | .19 (.09) | -3.85 (.37) | .12 (.16) | .45 [-.20, .44] | .85 (.12) | -.05 (.05) | .37 [-.16, .06] |
| PCI Q: 16 | .25 (.10) | -3.84 (.33) | .14 (.18) | .42 [-.21, .49] | .85 (.11) | -.06 (.06) | .33 [-.18, .06] |
| PCI Q: 17 | .38 (.12) | -3.43 (.36) | -.10 (.21) | .63 [-.51, .31] | .70 (.12) | .03 (.07) | .7 [-.11, .16] |

| | | | | | | | |
|---|---|---|---|---|---|---|---|
| PCI Q: 18 | .36 (.11) | -3.27 (.33) | -.21 (.16) | .2 [-.52, .11] | .65 (.11) | .05 (.05) | .31 [-.05, .16] |
| PCI Q: 19 | .19 (.11) | -3.69 (.34) | .04 (.16) | .81 [-.27, .35] | .76 (.11) | -.01 (.05) | .86 [-.11, .09] |
| PCI Q: 20 | .03 (.10) | -3.63 (.37) | -.01 (.16) | .93 [-.32, .29] | .77 (.12) | -.01 (.05) | .9 [-.11, .10] |
| PCI Q: 21 | .10 (.09) | -3.74 (.39) | .04 (.16) | .82 [-.28, .36] | .78 (.13) | -.01 (.05) | .85 [-.12, .10] |
| PCI Q: 22 | .14 (.10) | -3.63 (.44) | -.01 (.17) | .95 [-.34, .31] | .77 (.14) | -.00 (.05) | .95 [-.11, .10] |
| PCI Q: 23 | .25 (.09) | -3.38 (.29) | -.14 (.14) | .3 [-.41, .12] | .70 (.10) | .03 (.04) | .56 [-.06, .11] |
| PCI Q: 24 | .36 (.11) | -3.68 (.33) | .04 (.19) | .84 [-.34, .42] | .77 (.11) | -.01 (.06) | .9 [-.13, .12] |
| PCI Q: 25 | .25 (.10) | -3.86 (.34) | .17 (.19) | .37 [-.20, .53] | .78 (.11) | -.02 (.06) | .73 [-.15, .10] |
| PCI Q: 26 | .36 (.09) | -2.60 (.36) | -.45 (.15) | <.01 [-.76, -.15] | .47 (.12) | .13 (.05) | .01 [.03, .23] |
| PCI Q: 27 | .42 (.11) | -3.35 (.35) | -.14 (.21) | .5 [-.56, .27] | .62 (.12) | .08 (.07) | .25 [-.06, .23] |
| PCI Q: 28 | .45 (.10) | -3.00 (.32) | -.39 (.18) | .03 [-.74, -.04] | .53 (.11) | .14 (.06) | .02 [.02, .26] |
| PCI Q: 29 | .77 (.15) | -3.63 (.34) | -.02 (.24) | .94 [-.48, .44] | .81 (.11) | -.04 (.08) | .58 [-.20, .11] |
| PCI Q: 30 | .34 (.10) | -3.41 (.34) | -.12 (.14) | .4 [-.40, .16] | .68 (.11) | .04 (.05) | .46 [-.06, .13] |
| PCI Q: 31 | .58 (.13) | -3.40 (.31) | -.19 (.20) | .34 [-.59, .20] | .65 (.10) | .08 (.07) | .24 [-.05, .21] |
| PCI Q: 32 | .33 (.14) | -3.48 (.35) | -.14 (.23) | .55 [-.59, .32] | .67 (.12) | .07 (.08) | .35 [-.08, .22] |
| PCI Q: 33 | .49 (.13) | -3.26 (.31) | -.27 (.19) | .16 [-.64, .11] | .64 (.10) | .07 (.06) | .25 [-.05, .20] |
| PCI Q: 34a | .31 (.10) | -3.71 (.31) | .05 (.16) | .76 [-.26, .36] | .80 (.10) | -.03 (.05) | .51 [-.14, .07] |
| Total Score Transformation | .13 (.03) | -3.40 (.41) | -.03 (.05) | .5 [-.13, .06] | .67 (.13) | .01 (.02) | .52 [-.02, .04] |
| Total Score Distraction | .06 (.03) | -3.42 (.46) | -.02 (.04) | .6 [-.10, .06] | .69 (.15) | .01 (.01) | .69 [-.02, .03] |
| Total Score Reducting Demands | .13 (.05) | -2.75 (.44) | -.15 (.07) | .03 [-.29, -.02] | .49 (.14) | .04 (.02) | .05 [-.00, .09] |
| Total Score Retreating | .07 (.02) | -3.64 (.43) | -.00 (.04) | .97 [-.07, .07] | .71 (.14) | .00 (.01) | .75 [-.02, .03] |
| Total Score Worrying | .10 (.02) | -3.34 (.47) | -.02 (.03) | .49 [-.08, .04] | .72 (.16) | .00 (.01) | .84 [-.02, .02] |
| Total Score Resting | .14 (.04) | -1.92 (.52) | -.19 (.05) | <.01 [-.29, -.08] | .27 (.17) | .05 (.02) | <.01 [.02, .09] |
| Total Score Active Coping | .06 (.02) | -2.94 (.56) | -.03 (.02) | .19 [-.07, .01] | .54 (.19) | .01 (.01) | .25 [-.01, .02] |
| Total Score Passive Coping | .05 (.01) | -2.88 (.57) | -.02 (.02) | .16 [-.05, .01] | .54 (.19) | .01 (.01) | .27 [-.00, .02] |
| PCS Q: 1 | .36 (.11) | -3.81 (.16) | .37 (.20) | .06 [-.02, .75] | .78 (.06) | -.06 (.07) | .38 [-.19, .07] |
| PCS Q: 2 | .65 (.10) | -3.59 (.17) | -.04 (.18) | .82 [-.39, .31] | .73 (.06) | .04 (.06) | .56 [-.08, .16] |
| PCS Q: 3 | .63 (.10) | -3.55 (.17) | -.06 (.19) | .76 [-.43, .31] | .72 (.06) | .04 (.06) | .5 [-.08, .17] |
| PCS Q: 4 | .27 (.09) | -3.78 (.18) | .20 (.16) | .21 [-.11, .52] | .78 (.06) | -.03 (.06) | .6 [-.14, .08] |
| PCS Q: 5 | .45 (.10) | -3.62 (.19) | -.03 (.15) | .84 [-.32, .26] | .77 (.06) | -.03 (.05) | .57 [-.12, .07] |
| PCS Q: 6 | .41 (.10) | -3.49 (.17) | -.24 (.17) | .16 [-.57, .10] | .70 (.06) | .09 (.06) | .1 [-.02, .21] |
| PCS Q: 7 | .38 (.07) | -3.63 (.21) | .02 (.12) | .84 [-.22, .26] | .75 (.07) | .00 (.04) | .95 [-.08, .08] |
| PCS Q: 8 | .25 (.09) | -3.57 (.17) | -.06 (.16) | .7 [-.38, .26] | .72 (.06) | .04 (.06) | .44 [-.07, .15] |
| PCS Q: 9 | .41 (.09) | -3.66 (.17) | .17 (.18) | .34 [-.18, .52] | .76 (.06) | -.03 (.06) | .63 [-.15, .09] |
| Total Score PCS | .12 (.02) | -3.59 (.20) | .00 (.03) | .96 [-.06, .07] | .73 (.06) | .00 (.01) | .7 [-.02, .03] |
| IPQ-K Q: 2 Duration beliefs | .12 (.03) | -4.75 (.26) | .26 (.05) | <.01 [.16, .37] | 1.04 (.08) | -.07 (.02) | <.01 [-.10, -.04] |
| IPQ-K Q: 4 Treatment beliefs | .09 (.05) | -2.46 (.61) | -.15 (.08) | .05 [-.30, -.00] | .57 (.20) | .02 (.02) | .37 [-.03, .07] |
| Therapeutic relation | .06 (.06) | -2.15 (.89) | -.17 (.10) | .09 [-.37, .03] | .50 (.30) | .03 (.03) | .39 [-.04, .10] |
| IPQ-K Q: 6 Concerns | .19 (.03) | -4.00 (.24) | .12 (.06) | .03 [.01, .23] | .86 (.08) | -.02 (.02) | .31 [-.06, .02] |
| IPQ-K Q 7 Understanding | .00 (.04) | -3.39 (.42) | -.04 (.06) | .54 [-.16, .09] | .71 (.14) | .01 (.02) | .7 [-.03, .05] |
| FABQ Q: 1 | .07 (.03) | -3.58 (.21) | -.02 (.06) | .67 [-.14, .09] | .74 (.07) | .01 (.02) | .71 [-.03, .05] |
| FABQ Q: 2 | .04 (.03) | -3.58 (.23) | -.02 (.05) | .62 [-.12, .07] | .74 (.08) | .01 (.02) | .6 [-.02, .04] |
| Total Score FABQ | .05 (.02) | -3.72 (.23) | .01 (.03) | .75 [-.05, .06] | .77 (.08) | -.00 (.01) | .71 [-.02, .01] |
| Tampa Q: 1 | .03 (.12) | -4.02 (.33) | .29 (.22) | .18 [-.14, .72] | .85 (.11) | -.07 (.07) | .33 [-.22, .07] |
| Tampa Q: 2 | .23 (.10) | -3.64 (.32) | .02 (.18) | .92 [-.34, .37] | .72 (.11) | .03 (.06) | .68 [-.10, .15] |
| Tampa Q: 3 | .15 (.13) | -3.84 (.35) | .17 (.25) | .5 [-.31, .65] | .82 (.12) | -.05 (.08) | .57 [-.21, .12] |
| Tampa Q: 4 | .23 (.12) | -3.96 (.34) | .25 (.23) | .27 [-.20, .69] | .78 (.11) | -.02 (.08) | .85 [-.17, .14] |
| Tampa Q: 5 | -.06 (.17) | -4.26 (.39) | .55 (.31) | .07 [-.05, 1.15] | .87 (.13) | -.11 (.10) | .29 [-.31, .09] |
| Tampa Q: 6 | .13 (.10) | -3.80 (.32) | .13 (.18) | .48 [-.23, .49] | .78 (.11) | -.03 (.06) | .68 [-.15, .10] |
| Tampa Q: 7 | .25 (.09) | -3.37 (.33) | -.13 (.15) | .39 [-.43, .17] | .66 (.11) | .05 (.05) | .35 [-.05, .15] |
| Tampa Q: 8 | .23 (.17) | -4.09 (.37) | .40 (.29) | .17 [-.17, .97] | .85 (.12) | -.08 (.10) | .4 [-.27, .11] |
| Tampa Q: 9 | .17 (.09) | -3.10 (.34) | -.27 (.16) | .08 [-.58, .04] | .53 (.11) | .12 (.05) | .03 [.01, .22] |
| Tampa Q: 10 | .06 (.13) | -4.02 (.32) | .30 (.23) | .19 [-.15, .74] | .78 (.11) | -.02 (.08) | .82 [-.18, .14] |
| Tampa Q: 11 | -.08 (.12) | -4.10 (.33) | .33 (.21) | .12 [-.08, .74] | .84 (.11) | -.06 (.07) | .43 [-.19, .08] |
| Total Score Tampa | .04 (.02) | -4.29 (.50) | .04 (.03) | .16 [-.02, .10] | .86 (.17) | -.01 (.01) | .52 [-.03, .01] |
| PSEQ Q: 1 | -.31 (.07) | -2.26 (.74) | .03 (.14) | .82 [-.24, .31] | .52 (.26) | .00 (.05) | .97 [-.09, .10] |
| PSEQ Q: 2 | -.31 (.06) | -2.06 (.72) | .00 (.13) | .98 [-.26, .27] | .52 (.25) | -.00 (.05) | .98 [-.09, .09] |
| Total Score PSEQ | -.18 (.04) | -4.90 (.65) | .12 (.06) | .05 [.00, .24] | 1.10 (.22) | -.03 (.02) | .1 [-.07, .01] |
| PVAQ Q: 1 | .15 (.07) | -3.62 (.21) | -.01 (.12) | .97 [-.24, .23] | .70 (.07) | .05 (.04) | .2 [-.03, .13] |
| PVAQ Q: 2 | .13 (.06) | -3.79 (.29) | .08 (.11) | .47 [-.13, .29] | .75 (.09) | .00 (.04) | .93 [-.07, .07] |
| PVAQ Q: 3 | .25 (.07) | -3.54 (.34) | -.00 (.11) | .96 [-.22, .21] | .71 (.11) | .02 (.04) | .67 [-.06, .09] |
| PVAQ Q: 4 | .07 (.06) | -3.13 (.30) | -.19 (.10) | .06 [-.39, .01] | .59 (.10) | .06 (.03) | .06 [-.00, .13] |
| PVAQ Q: 5 | .08 (.06) | -3.23 (.31) | -.15 (.10) | .14 [-.35, .05] | .58 (.10) | .07 (.03) | .04 [.00, .14] |
| PVAQ Q: 6 | .15 (.07) | -3.61 (.21) | .00 (.12) | .99 [-.23, .23] | .73 (.07) | .02 (.04) | .63 [-.06, .10] |
| PVAQ Q: 7 | .35 (.06) | -3.07 (.23) | -.25 (.11) | .02 [-.46, -.05] | .58 (.08) | .09 (.04) | <.01 [.03, .16] |
| PVAQ Q: 8 | -.03 (.06) | -3.76 (.30) | .05 (.11) | .63 [-.16, .26] | .75 (.10) | .00 (.04) | .93 [-.07, .07] |
| PVAQ Q: 9 | .08 (.06) | -3.66 (.28) | .02 (.11) | .88 [-.20, .23] | .71 (.09) | .03 (.04) | .43 [-.04, .10] |
| PVAQ Q: 10 | .09 (.07) | -3.59 (.19) | -.04 (.12) | .74 [-.27, .19] | .73 (.06) | .03 (.04) | .43 [-.05, .11] |

| | | | | | | | |
|---|---|---|---|---|---|---|---|
| PVAQ Q: 11 | .13 (.06) | -3.64 (.25) | -.00 (.10) | .98 [-.21, .20] | .76 (.08) | .00 (.03) | .9 [-.06, .07] |
| PVAQ Q: 12 | .15 (.07) | -3.63 (.18) | -.03 (.12) | .8 [-.28, .21] | .74 (.06) | .03 (.04) | .48 [-.05, .11] |
| PVAQ Q: 13 | .17 (.08) | -3.61 (.18) | -.04 (.14) | .75 [-.32, .23] | .73 (.06) | .03 (.05) | .46 [-.06, .13] |
| PVAQ Q: 14 | .18 (.07) | -3.47 (.19) | -.14 (.12) | .23 [-.37, .09] | .70 (.06) | .05 (.04) | .21 [-.03, .13] |
| PVAQ Q: 15 | .37 (.07) | -3.52 (.18) | -.05 (.13) | .72 [-.30, .21] | .73 (.06) | .01 (.04) | .85 [-.08, .10] |
| PVAQ Q: 16 | .04 (.05) | -3.64 (.28) | .01 (.09) | .95 [-.18, .19] | .78 (.09) | -.01 (.03) | .75 [-.07, .05] |
| Total Score PVAQ | .02 (.01) | -3.34 (.40) | -.01 (.01) | .41 [-.03, .01] | .69 (.13) | .00 (.00) | .65 [-.01, .01] |
| DASS-21 Dep Q: 1 | .40 (.13) | -3.55 (.17) | -.24 (.24) | .32 [-.72, .24] | .72 (.06) | .12 (.08) | .16 [-.05, .28] |
| DASS-21 Dis Q: 1 | .25 (.14) | -3.60 (.16) | -.08 (.25) | .75 [-.56, .40] | .73 (.05) | .10 (.08) | .25 [-.07, .26] |
| DASS-21 Dep Q: 2 | .17 (.12) | -3.60 (.17) | -.06 (.20) | .76 [-.46, .34] | .73 (.06) | .06 (.07) | .36 [-.07, .20] |
| DASS-21 Dis Q: 2 | .11 (.13) | -3.70 (.16) | .21 (.24) | .36 [-.25, .67] | .76 (.06) | -.03 (.08) | .68 [-.19, .12] |
| DASS-21 Dep Q: 3 | .16 (.14) | -3.66 (.16) | .07 (.24) | .77 [-.41, .55] | .76 (.05) | -.01 (.08) | .88 [-.17, .15] |
| DASS-21 Dis Q: 3 | .23 (.12) | -3.52 (.17) | -.27 (.20) | .19 [-.67, .13] | .70 (.06) | .14 (.07) | .05 [.00, .27] |
| DASS-21 Dep Q: 4 | .09 (.12) | -3.60 (.17) | -.09 (.20) | .67 [-.48, .31] | .72 (.06) | .09 (.07) | .19 [-.04, .23] |
| DASS-21 Dis Q: 4 | .19 (.10) | -3.60 (.19) | -.01 (.18) | .95 [-.36, .34] | .73 (.06) | .04 (.06) | .5 [-.08, .16] |
| DASS-21 Dep Q: 5 | .15 (.14) | -3.55 (.16) | -.25 (.24) | .31 [-.72, .23] | .70 (.05) | .16 (.08) | .05 [-.00, .32] |
| DASS-21 Dis Q: 5 | .22 (.09) | -3.64 (.21) | .08 (.16) | .62 [-.23, .38] | .73 (.07) | .02 (.05) | .7 [-.08, .12] |
| DASS-21 Dep Q: 6 | .12 (.14) | -3.64 (.16) | -.08 (.25) | .76 [-.57, .41] | .74 (.05) | .09 (.09) | .31 [-.08, .26] |
| DASS-21 Dis Q: 6 | .15 (.11) | -3.60 (.17) | -.05 (.21) | .8 [-.46, .35] | .73 (.06) | .07 (.07) | .33 [-.07, .21] |
| DASS-21 Dep Q: 7 | .22 (.21) | -3.63 (.15) | .08 (.33) | .81 [-.56, .72] | .74 (.05) | .04 (.11) | .68 [-.16, .25] |
| DASS-21 Dis Q: 7 | .23 (.11) | -3.59 (.18) | -.08 (.19) | .68 [-.45, .29] | .72 (.06) | .09 (.07) | .19 [-.04, .22] |
| Total Score DASS-21 Dep | .07 (.03) | -3.64 (.17) | -.01 (.04) | .86 [-.09, .07] | .73 (.06) | .01 (.01) | .52 [-.02, .04] |
| Total Score DASS-21 DIS | .07 (.02) | -3.83 (.20) | .04 (.03) | .25 [-.03, .11] | .80 (.07) | -.01 (.01) | .37 [-.03, .01] |
| PHQ Q: 1 | .03 (.11) | -3.75 (.17) | .21 (.19) | .27 [-.17, .60] | .77 (.06) | .00 (.07) | .97 [-.13, .13] |
| PHQ Q: 2 | .14 (.12) | -3.63 (.17) | -.02 (.21) | .93 [-.42, .39] | .74 (.06) | .06 (.07) | .35 [-.07, .20] |
| Total Score PHQ | .04 (.07) | -3.80 (.17) | .14 (.10) | .16 [-.05, .34] | .78 (.06) | -.03 (.03) | .34 [-.10, .03] |

BMI: Body Mass Index, dich: dichotomized outcome, NPRS: numeric pain rating scale, NNGB: Nederlandse Norm Gezond Bewegen (Dutch Healthy Exercise Norm) PDI: Pain Disability Index, PCI: Pain Coping Inventory, Q: Question, B-IPQ: Brief Illness Perception Questionnaire, IPQ-K: Illness Perception Questionnaire-Kort, TSK-11: Tampa Scale of Kinesiofobia 11-item version, PSEQ-2: Pain Self-efficacy Questionnaire 2-item short form, PVAQ: Pain Vigilance Awareness Questionnaire, DASS-21: Depression Anxiety Stress Scales – Short Form, Dep: Depression subscale, Dis: Distress subscale, PHQ: Patient Health Questionnaire (short version). (For a more detailed description of the questionnaires and items used, see: A study protocol for the validation of a prognostic model with an emphasis on modifiable factors to predict chronic pain after a new episode of acute- or subacute nonspecific idiopathic, non-traumatic neck pain presenting in primary care | PLOS One
