# Supplementary material for: Clinical indicators associated with pain trajectories in non-specific neck pain: a secondary analysis of a multicentre prospective observational cohort to develop a clinical compass for personalised physiotherapy in primary care
Source: BMJ Open. 2026 Aug 3;16(8):e115274. doi: 10.1136/bmjopen-2025-115274 (PMC13435912; doi:10.1136/bmjopen-2025-115274)
Supplement: Supplementary file 4 [file bmjopen-16-8-s004.pdf]

Appendix 4

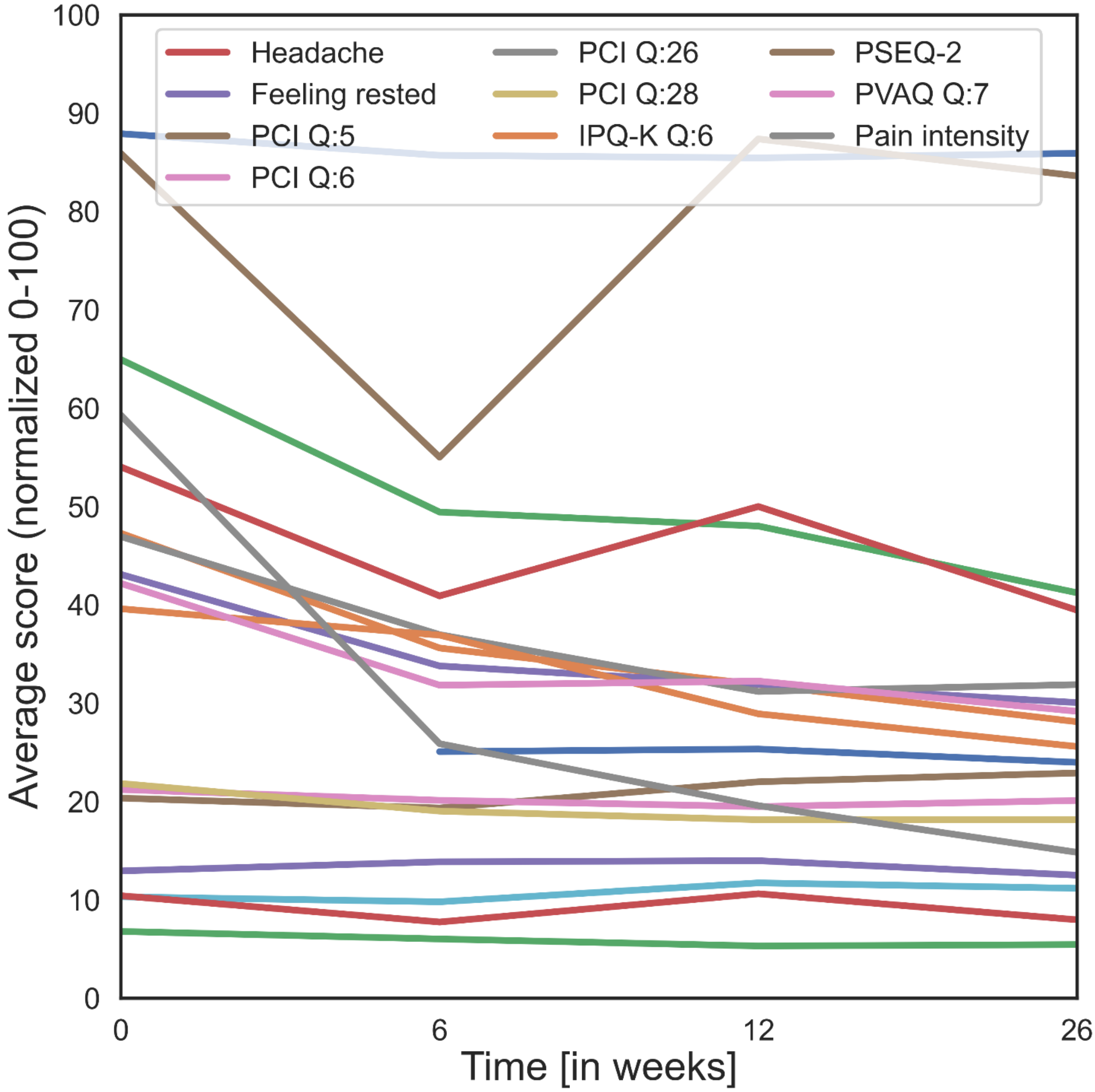

Visualization of the mean outcome of the significant clinical indicators over time, after min-max normalization (per indicators). In general, the clinical indicators show a downward trend; however, it is less steep than pain intensity. Only the clinical indicators measured at more than two time points are displayed. Abbreviations: PCI = Pain Coping Inventory; IPQ-k: Illness Perception Questionnaire – kort (short form); TSK = Tampa Scale for Kinesiophobia; PVAQ = Pain Vigilance and Awareness Questionnaire.
