# Supplementary material for: Clinical indicators associated with pain trajectories in non-specific neck pain: a secondary analysis of a multicentre prospective observational cohort to develop a clinical compass for personalised physiotherapy in primary care
Source: BMJ Open. 2026 Aug 3;16(8):e115274. doi: 10.1136/bmjopen-2025-115274 (PMC13435912; doi:10.1136/bmjopen-2025-115274)
Supplement: Supplementary file 5 [file bmjopen-2025-115274.reviewer_comments.pdf]

PEER REVIEW HISTORY

BMJ Open publishes all reviews undertaken for accepted manuscripts. Reviewers are asked to complete a checklist review form and are provided with free text boxes to elaborate on their assessment. These free text comments are reproduced below.

ARTICLE DETAILS

Title (Provisional)

Clinical indicators associated with pain trajectories in non-specific neck pain: a secondary analysis of a multicenter prospective observational cohort to develop a clinical compass for personalized physiotherapy in primary care.

Authors

Verwoerd, Martine J.; Felius, Richard A.W.; Konings, Sophie; Kiers, Henri; Smeets, Rob. J.E.M.

VERSION 1 - REVIEW

**Reviewer**	**1**
**Name**	**Reddy, Ravi**
**Affiliation**	**King Khalid University, Department of Medical Rehabilitation Sciences, King Khalid University, Abha, Saudi Arabia**
**Date**	**11-Feb-2026**
**COI**	**None**

Thank you for the opportunity to review this manuscript. The topic is highly relevant to primary care physiotherapy, and the attempt to move from broad prognostic stratification toward individualized clinical profiling is timely. In the spirit of strengthening the manuscript, I outline below several areas where further clarification, methodological refinement, and conceptual precision are needed. The comments focus exclusively on aspects that require improvement or further justification.

In the abstract, the objectives remain somewhat conceptually vague. The term “modifiable prognostic factors” is used without clarifying how modifiability was operationally defined in the context of this analysis, especially given that some factors were only measured at baseline and not manipulated. The abstract also does not specify whether the identified indicators were baseline predictors, time-varying covariates, or both, which is essential for understanding the nature of the associations. In the methods section of the abstract, it is unclear how many candidate indicators were tested and whether any correction for multiple testing was applied, despite the very large number of examined variables. The results section does not report effect sizes or the magnitude of explained variance, making it

difficult to judge the clinical relevance of the findings. The statement that 21 indicators were significant but 19 were retained after excluding two for collinearity lacks detail about the decision rules applied. Finally, the conclusion overstates practical applicability without acknowledging that the compass has not yet undergone external validation, impact analysis, or usability testing; this limitation should be reflected already in the abstract.

In the introduction, the rationale for shifting from existing stratification tools (e.g., STarT MSK) to a single-item, data-driven "clinical compass" requires deeper theoretical justification. The manuscript assumes that single items may be more practical and equally or more informative than validated composite scales, but this assumption is not sufficiently grounded in psychometric theory. The conceptual distinction between prediction, prognostic association, and treatment targeting remains blurred. While the authors refer to "modifiable prognostic factors," it is not made explicit whether the goal is prognostic enrichment, mechanism identification, or intervention guidance. The introduction would benefit from a clearer explanation of why pain intensity trajectories were chosen as the sole outcome rather than disability or global recovery, especially since pain disability is included as a predictor. Furthermore, the clinical problem of transition from acute to chronic pain is mentioned, but the study does not explicitly model chronicity as an outcome, creating a mismatch between the stated clinical problem and the analytical strategy. The novelty claim would be strengthened by more explicitly contrasting this work with prior multivariable prognostic models developed from the same cohort and clarifying how this analysis advances beyond them methodologically and conceptually.

In the methods section, several aspects require clarification and additional rigor. First, the handling of missing data is insufficiently described. Although linear mixed models can handle missing outcome data under a missing-at-random assumption, the manuscript does not report the extent of missingness per variable, nor does it justify the plausibility of the missing-at-random assumption. Second, the modelling strategy involves testing 143 clinical indicators individually as effect modifiers. This univariable screening approach raises substantial concerns about multiplicity and false-positive findings, yet no correction strategy (e.g., false discovery rate control, Bonferroni adjustment, shrinkage methods) is described. Relying on $p < 0.05$ without adjustment substantially inflates type I error. Third, adding each indicator separately precludes assessment of independent effects in a multivariable context. The resulting compass therefore reflects marginal rather than mutually adjusted associations, which should be acknowledged and methodologically addressed. Fourth, the decision to retain indicators based partly on "clinical expertise and practical considerations" when collinearity exceeded $|r| \geq 0.70$ introduces subjectivity; a more transparent, predefined rule is needed. Fifth, the choice to model only random intercepts without random slopes may inadequately capture inter-individual variability in trajectories; justification for excluding random slopes is not provided. Sixth, time is treated as 0, 6, 12, and 24 weeks, but the scaling of the time variable (e.g., centered, rescaled) is not reported, which affects interpretability of coefficients. Seventh, the marginal R^2 values reported in

Table 3 are not clearly defined (e.g., incremental versus total explained variance), and the method used to compute them in mixed models should be specified. Finally, the rationale for standardizing all indicators to z-scores for visualization, while useful for comparison, may obscure clinical meaning; no justification is provided for using sample-based standardization rather than clinically anchored thresholds.

In the results section, the reporting lacks sufficient quantitative detail to judge clinical relevance. Although the quadratic model is statistically superior, the practical difference between linear and quadratic fits is not quantified in terms of effect size or explained variance. The coefficients for time in Table 2 differ between the linear-only and quadratic models, but the interpretation of these differences is not discussed. For the significant clinical indicators, only p-values and coefficients are presented; confidence intervals and effect magnitudes are insufficiently contextualized in terms of clinical impact. There is no information on how many indicators were significant for the linear term only, quadratic term only, or both, nor how these were prioritized in the compass. The R^2 values reported appear very similar across indicators (around 0.45–0.49), raising questions about how much incremental variance each indicator contributes. Furthermore, the baseline correlations used to assess collinearity were calculated only at T0, yet some variables were time-varying; the implications of this approach are not discussed. The participant flow diagram is relegated to an appendix, and attrition across time points is not described in the main text, limiting transparency. Finally, the radar chart visualization is presented without empirical evaluation of interpretability or user comprehension, and no example is provided demonstrating how different profiles correspond to different predicted trajectories.

In the discussion, causal language is occasionally implied despite the purely observational and associational design. Statements suggesting that targeting these indicators may prevent chronic pain are speculative and should be more clearly separated from empirical findings. The comparison with STarT MSK does not sufficiently acknowledge that the compass has not undergone validation for predictive accuracy, calibration, or discrimination, making direct comparison premature. The measurement limitations of single items are acknowledged, but the implications for measurement error propagation within mixed models are not elaborated. The discussion of type I and type II error remains somewhat reassuring in tone without presenting sensitivity analyses or robustness checks to substantiate confidence in the findings. Moreover, the clinical implications assume that modifying these indicators will alter pain trajectories, yet no mediation or temporal sequencing analyses were performed to support this assumption. The potential confounding effect of treatment variation across physiotherapists and practices is not addressed, even though therapeutic interventions were not standardized and could influence pain trajectories independently of baseline indicators.

In the conclusion, the statement that the compass provides a “clinically feasible method” is premature in the absence of usability testing, implementation research, and external validation. The conclusion should more clearly delimit the scope of inference to exploratory identification of candidate indicators rather than implying readiness for clinical deployment.

It would also be important to emphasize that the compass reflects associations with pain intensity trajectories in a specific Dutch primary care context and may not generalize without replication. Finally, the conclusion does not adequately reflect the substantial statistical uncertainty introduced by multiple testing and univariable screening.

**Reviewer**	**2**
**Name**	**Danazumi, Musa**
**Affiliation**	**La Trobe University Discipline of Physiotherapy, Physiotherapy**
**Date**	**19-Feb-2026**
**COI**	**None**

Overall Recommendation: Minor Revision

This manuscript presents an innovative secondary analysis of a large prospective cohort exploring modifiable clinical indicators associated with pain intensity trajectories in individuals with (sub)acute non-specific neck pain. The study is clinically relevant, methodologically sound, and aligns well with contemporary efforts to operationalise personalised physiotherapy.

The longitudinal design, repeated measurements, biopsychosocial breadth of indicators, and use of growth-curve modelling are clear strengths. The development of the "clinical compass" as a translational tool is particularly novel and represents an important step toward bridging research and clinical reasoning.

Overall, the manuscript is of high quality. I offer several suggestions that may help strengthen clarity and interpretability.

Major Suggestions

1. Clarification of Exploratory Nature

Given the large number of clinical indicators evaluated, it may be helpful to explicitly frame the analysis as exploratory and hypothesis-generating. While adjustment for multiple testing is not always required in exploratory analyses, a brief justification of this decision would improve transparency.

This would strengthen the methodological clarity without altering the study's conclusions.

2. Modelling Strategy Transparency

Each modifiable indicator appears to have been added separately to the growth model. Clarifying that this represents a screening or effect-modifier exploration approach (rather than a fully adjusted multivariable prediction model) would help readers interpret the findings appropriately.

It may also be useful to briefly acknowledge that interrelationships between predictors were not modelled simultaneously.

3. Time-Varying Indicators

Some indicators were measured repeatedly. A short clarification on whether these were treated as time-varying covariates, and how temporal ordering was handled, would enhance methodological transparency.

Even a concise statement in the Methods would be sufficient.

4. Interpretation of Marginal R^2

The use of marginal R^2 to rank indicator relevance is helpful. Providing a brief description of how R^2 was calculated and clarifying that it reflects variance explained within the modelling framework would improve interpretability.

5. Clinical Compass Interpretation

The clinical compass is a highly innovative feature of the manuscript. Since it is based on standardized z-scores relative to the cohort mean, it may be helpful to emphasize in the Discussion that this tool currently supports structured clinical reasoning rather than providing prescriptive thresholds.

A short forward-looking statement about future validation would further strengthen the translational message.

Minor Suggestions

• Brief clarification of missing data assumptions (e.g., MAR in LMM).

• Report attrition at each time point.

VERSION 1 - AUTHOR RESPONSE

Reviewer: 1
Dr. Ravi Reddy, King Khalid University
Comments to the Author:
Thank you for the opportunity to review this manuscript. The topic is highly relevant to primary care physiotherapy, and the attempt to move from broad prognostic stratification toward individualized clinical profiling is timely. In the spirit of strengthening the manuscript, I outline below several areas where further clarification, methodological refinement, and conceptual precision are needed. The comments focus exclusively on aspects that require improvement or further justification.

Abstract

1. ***In the abstract, the objectives remain somewhat conceptually vague. The term "modifiable prognostic factors" is used without clarifying how***

modifiability was operationally defined in the context of this analysis, especially given that some factors were only measured at baseline and not manipulated.

Response:

We agree that greater clarity is needed regarding the use of the term *modifiable prognostic factors*. In the underlying cohort study, potential modifiability (by an intervention) was identified *a priori* based on previous literature and expert consensus obtained through a Delphi procedure (see references cited below). In the present secondary analysis, modifiability was therefore not empirically tested, but conceptually informed by this prior work.

To address this, we revised the abstract objective to *"clinical indicators reflecting potentially modifiable prognostic factors."* We also clarified in the Introduction how the potentially modifiable prognostic factors are defined in the present study.

Revisions made:

Abstract, Objectives revised to

"To identify clinical indicators reflecting potentially treatment-modifiable prognostic factors associated with pain recovery in patients with non-specific neck pain..."

Introduction: added the conceptual definition:
"Potentially modifiable prognostic factors are defined here as patient characteristics that may be amenable through treatment *to change and associated with subsequent pain trajectories."*

Literature:

Verwoerd M, Wittink H, Maissan F, Smeets R. Consensus of potential modifiable prognostic factors for persistent pain after a first episode of nonspecific idiopathic, non-traumatic neck pain: results of nominal group and Delphi technique approach. BMC Musculoskelet Disord. 2020 Oct 7;21(1):656. doi: 10.1186/s12891-020-03682-8.

Verwoerd M, Wittink H, Maissan F, de Raaij E, Smeets RJEM. Prognostic factors for persistent pain after a first episode of nonspecific idiopathic, non-traumatic neck pain: A systematic review. Musculoskelet Sci Pract. 2019 Jul;42:13-37. doi: 10.1016/j.msksp.2019.03.009. Epub 2019 Apr 2. PMID: 31026716.

2. ***The abstract also does not specify whether the identified indicators were baseline predictors, time-varying covariates, or both, which is essential for understanding the nature of the associations.***

Response:

We revised the Methods section of the abstract to clarify that some indicators were assessed only at baseline, whereas others were assessed repeatedly and examined accordingly in relation to pain trajectories. This distinction was already specified in Table 1 of the manuscript and is now also explicitly reflected in the abstract.

Revisions made:

Abstract, Primary and secondary outcome measures: added
"Baseline and repeated indicators were examined as effect modifiers of pain trajectories using growth-curve models."

3. ***In the methods section of the abstract, it is unclear how many candidate indicators were tested and whether any correction for multiple testing was applied, despite the very large number of examined variables. The results section does not report effect sizes or the magnitude of explained variance, making it difficult to judge the clinical relevance of the findings.***

Response:

We agree that the abstract needs to better reflect the analytic strategy. We clarified that questionnaire total scores and individual questionnaire items reflecting selected potentially modifiable factors were tested separately as candidate indicators. No formal correction for multiple testing was applied because the analysis was exploratory and intended to identify candidate indicators for inclusion in a prototype clinical compass.

Given the abstract word limit and the large number of separately tested indicators, we did not include full details on all candidate indicators or marginal R^2 values in the abstract, but we report these in the main manuscript and now explicitly frame the analyses as exploratory in the Discussion.

Revisions made:

Abstract, Primary and secondary outcome measures: revised to
"Questionnaire's total scores and individual items reflecting selected potentially modifiable factors were tested separately as candidate indicators."

4. ***The statement that 21 indicators were significant but 19 were retained after excluding two for collinearity lacks detail about the decision rules applied.***

Response:

We clarified the decision-making process in the Methods and made the abstract more explicit. Specifically, collinearity among clinical indicators was assessed using

Pearson's correlation coefficient, with overlap defined *a priori* as $|\ r\ | \geq 0.70$. Indicators showing such overlap were consolidated or removed to reduce redundancy and respondent burden, while maintaining the applicability and feasibility of the clinical compass for routine clinical use. However, due to abstract word limits, this level of detail could not be fully reported there.

Revisions made:

Abstract, Results: revised to
"Two characteristics were excluded as they demonstrated high intercorrelation."

5. ***Finally, the conclusion overstates practical applicability without acknowledging that the compass has not yet undergone external validation, impact analysis, or usability testing; this limitation should be reflected already in the abstract.***

Response:
We fully agree. We revised the abstract conclusion to better reflect the prototype status of the clinical compass and the need for further evaluation before implementation. Given the abstract's word limit, we phrased this concisely and cautiously. We also expanded the Discussion to make the required future steps more explicit. See also our response to the comments 29-31.

Revisions made:

Abstract, Conclusion: revised to
"This study provides a prototype clinical compass that may inform prognostic reasoning in primary care physiotherapy, but further evaluation is needed before clinical implementation."

Introduction

6. ***In the introduction, the rationale for shifting from existing stratification tools (e.g., STarT MSK) to a single-item, data-driven "clinical compass" requires deeper theoretical justification. The manuscript assumes that single items may be more practical and equally or more informative than validated composite scales, but this assumption is not sufficiently grounded in psychometric theory.***

Response:
We agree that the earlier version was too strongly phrased. We revised the Introduction to clarify that single-item clinical indicators are used here as pragmatic signals of potentially relevant domains, while full questionnaires remain relevant for measuring broader constructs. This better positions the clinical compass as a profiling aid rather than a replacement for validated instruments.

Revisions made:

Introduction: added and revised the following text:
"The present study examines whether selected single-item clinical indicators from validated questionnaires can function as pragmatic signals of potentially relevant modifiable domains in routine primary care. These indicators are intended to support focused assessment and prognostic reasoning, while full questionnaires remain relevant for measuring broader underlying constructs."

7. ***The conceptual distinction between prediction, prognostic association, and treatment targeting remains blurred. While the authors refer to "modifiable prognostic factors," it is not made explicit whether the goal is prognostic enrichment, mechanism identification, or intervention guidance.***

Response:
We agree that the distinction between prediction, prognostic association, and treatment targeting needs to be made more explicit. We revised the Introduction to sharpen this distinction. The present study is framed as an examination of associations between potentially modifiable clinical indicators and pain trajectories, with the purpose of supporting prognostic reasoning and focused assessment. We also adjusted the Discussion to use more cautious language regarding clinical implications. We would also like to refer to our response to comments 18 and 28.

Responses made:

Introduction:

Clarified the conceptual framing by adding:
"Potentially modifiable prognostic factors are defined here as patient characteristics that may be amenable to change and associated with subsequent pain trajectories."

"Potentially modifiable prognostic factors are defined here as patient characteristics that may be amenable to change through treatment and associated with subsequent pain trajectories. The present study examines whether selected single-item clinical indicators from validated questionnaires can function as pragmatic signals of potentially relevant modifiable domains in routine primary care. These indicators are intended to support focused assessment and prognostic reasoning, while full questionnaires remain relevant for measuring broader underlying constructs. Moving beyond broad stratification towards a more personalized and data-driven approach may therefore enhance the clinical usefulness of prognostic information in primary care physiotherapy. A concise clinical compass based on empirically selected clinical indicators may support patient-specific profiling while remaining feasible in routine care. Examining combinations of clinical indicators, rather than isolated factors alone, may provide a more informative basis for prognostic reasoning and individualized assessment."

8. ***The introduction would benefit from a clearer explanation of why pain intensity trajectories were chosen as the sole outcome rather than disability or global recovery, especially since pain disability is included as a predictor.***

Response:

We agree and now clarify this point more explicitly in the manuscript. Pain intensity was selected as the outcome because it was the primary variable assessed repeatedly across follow-up in the original cohort, allowing recovery to be modeled as a longitudinal trajectory. Other clinically relevant outcomes, such as disability or global recovery, were not available with the same repeated measurement structure and were therefore not suitable for comparable trajectory analyses in the present study. We also acknowledge in the Discussion that these outcomes may capture complementary aspects of recovery and should be considered in future research.

Revisions made:

Introduction: added
"Pain intensity was selected as the outcome because it was the primary variable assessed repeatedly across follow-up in the original cohort, allowing recovery to be modeled as a dynamic process over time."

Discussion: added:

"Relatedly, outcome selection was constrained by the cohort's repeated-measures structure. Comparable longitudinal data were not available for other clinically relevant outcomes, such as disability or global recovery, and these outcomes could therefore not be examined using similar trajectory analyses in the present study. Future longitudinal research should examine whether these outcomes provide complementary insights into recovery trajectories."

9. ***Furthermore, the clinical problem of transition from acute to chronic pain is mentioned, but the study does not explicitly model chronicity as an outcome, creating a mismatch between the stated clinical problem and the analytical strategy.***

Response:
We revised the Introduction to better align the clinical motivation with the analytical strategy. The text now emphasizes that the study takes a trajectory-based perspective on recovery over time rather than directly modeling chronicity as a dichotomous outcome. This choice was made because modeling pain trajectories better captures the heterogeneity of recovery processes and provides more nuanced information than a simple chronic versus non-chronic classification. We have clarified this rationale in the Introduction.

Revisions made:

Introduction: clarified the trajectory-based framing in the final paragraph:
"By focusing on pain intensity trajectories, recovery was approached as a dynamic process over time."

10. ***The novelty claim would be strengthened by more explicitly contrasting this work with prior multivariable prognostic models developed from the same cohort and clarifying how this analysis advances beyond them methodologically and conceptually.***

Response:
We revised the Introduction to make this distinction more explicit. The revised text now directly contrasts the earlier multivariable baseline prognostic model for chronic pain with the present trajectory-based, more practice-oriented analysis focused on questionnaire-level and item-level indicators and their translation into a visual patient-specific profile.

Revisions made:

Introduction: added
"This analysis extends previous work from the same cohort, which focused on developing a multivariable prognostic model for chronic pain as a dichotomous outcome. The present study takes a more practice-oriented, trajectory-based approach by examining questionnaire-level and item-level clinical indicators of pain intensity over time and translating nonredundant indicators into a patient-specific visual profile."

Method
In the methods section, several aspects require clarification and additional rigor.

11. ***First, the handling of missing data is insufficiently described. Although linear mixed models can handle missing outcome data under a missing-at-random assumption, the manuscript does not report the extent of missingness per variable, nor does it justify the plausibility of the missing-at-random assumption.***

Response:

We agree that the handling of missing data was insufficiently described. We therefore expanded the Methods section and added an appendix table reporting data availability per variable and time point. We also clarified that the linear mixed models were estimated using all available observations under maximum likelihood, which retains participants with incomplete repeated outcome data under a missing-at-random assumption. We now describe this assumption more explicitly and cautiously.

Revisions made:

Method added:

“Linear mixed models incorporate both fixed effects, representing average changes across individuals, and random effects, which account for individual variability. They also allow the inclusion of all available repeated observations, so that participants with incomplete follow-up data can be retained under the assumption that data are missing at random. Data availability per variable and measurement occasion is reported in Appendix 2.”

Results added:

“At follow-up, data were available for 449 participants at 6 weeks, 379 at 3 months, and 391 at 6 months. Only 78 participants did not complete any follow-up measurement. Participant flow is presented in Appendix 1, participant characteristics in Table 1, and data availability per variable and measurement occasion in Appendix 2.”

12. ***Second, the modelling strategy involves testing 143 clinical indicators individually as effect modifiers. This univariable screening approach raises substantial concerns about multiplicity and false-positive findings, yet no correction strategy (e.g., false discovery rate control, Bonferroni adjustment, shrinkage methods) is described. Relying on $p < 0.05$ without adjustment substantially inflates type I error.***

Response:

This is a valid methodological concern, as with 143 indicators tested individually, the expected number of false positives under a $p < 0.05$ threshold without correction is approximately seven, meaning some identified effect modifiers may reflect chance findings rather than true associations. We acknowledged this limitation explicitly in the Discussion section. However, our analytical approach was intentionally exploratory rather than confirmatory. All 143 variables were pre-selected based on theoretical and clinical relevance to pain trajectories, which constrains the risk of spurious findings compared to an unconstrained screen. We also note that applying stringent multiplicity corrections (e.g., Bonferroni) in this context carries a meaningful cost: given the moderate sample size, such corrections would substantially reduce statistical power and risk excluding clinically important effect modifiers (type II error). As clarified in the Discussion, ongoing work is focusing on whether these indicators are comprehensible, responsive to change over time, and associated with relevant outcomes, to support further refinement of the clinical compass.

13. ***Third, adding each indicator separately precludes assessment of independent effects in a multivariable context. The resulting compass therefore reflects marginal rather than mutually adjusted associations, which should be acknowledged and methodologically addressed.***

Response:

Indeed, the compass reflects marginal associations rather than mutually adjusted effects, and we acknowledge that likely some identified modifiers capture overlapping rather than independent information. To partially address this, we employed a correlation matrix to identify and exclude highly redundant variables, reducing the risk that the compass contains multiple indicators with the same information. We decided not to use a multivariable model as it would likely yield fewer independent predictors at the cost of clinical granularity. Many of the included variables are not interchangeable as they reflect distinct patient characteristics (e.g., psychological, functional, and social domains) that a clinician may wish to consider individually when tailoring treatment. The compass is therefore best understood as an exploratory clinical decision-support tool that visualizes potentially relevant effect modifiers, rather than an optimal predictive model.

We made several revisions to more clearly position the tool as exploratory. Please see our response to comment 31 by Reviewer 1 and comments 1 and 3 by Reviewer 2.

14. ***Fourth, the decision to retain indicators based partly on "clinical expertise and practical considerations" when collinearity exceeded |r| ≥ 0.70 introduces subjectivity; a more transparent, predefined rule is needed.***

Response:

We agree that relying solely on clinical expertise and practical considerations may appear insufficiently transparent. We therefore revised the Methods to state more explicitly that, when collinearity exceeded |r|≥0.70, the final selection was guided by predefined criteria rather than ad hoc judgment. Specifically, preference was given to indicators that were significantly associated with changes in pain over time, actionable within physiotherapy practice, and non-redundant in content. Where overlapping indicators captured similar content, more specific and clinically informative items were retained over broader composite scores. These criteria are now explicitly described in the Methods section on collinearity among clinical indicators.

Revisions made:

"Where pairs of significant indicators exceeded this threshold, the retained indicator was selected based on predefined practical and clinical criteria, including strength of association with changes in pain over time, clinical actionability within physiotherapy practice, and non-redundancy in content. Where content overlapped, more specific and informative items were preferred over broader composite scores."

The following references were added to the text:

Wang, X., Andrinopoulou, E. R., Veen, K. M., Bogers, A. J. J. C., & Takkenberg, J. J. M. (2022). Statistical primer: an introduction to the application of linear mixed-effects models in cardiothoracic surgery outcomes research-a case study using homograft

pulmonary valve replacement data. European journal of cardio-thoracic surgery : official journal of the European Association for Cardio-thoracic Surgery, 62(4), ezac429. https://doi.org/10.1093/ejcts/ezac429

Yoo, W., Mayberry, R., Bae, S., Singh, K., Peter He, Q., & Lillard, J. W., Jr (2014). A Study of Effects of MultiCollinearity in the Multivariable Analysis. International journal of applied science and technology, 4(5), 9–19.

15. ***Fifth, the choice to model only random intercepts without random slopes may inadequately capture inter-individual variability in trajectories; justification for excluding random slopes is not provided.***

Response:

Random slopes were considered but not used for two reasons. First, with a maximum of four measurement occasions per individual, there are insufficient within-person observations to reliably estimate both a random intercept and slope. With so few repeated observations, reliably estimating both a random intercept and a random slope at the individual level is statistically challenging: the within-person data are insufficient to separate true slope variability from residual noise. Second, introducing random slopes in this context frequently causes model convergence failures given the data structure.

We acknowledge that this means inter-individual variability in rate of change is not explicitly modelled, and residualises some trajectory heterogeneity. Future studies with more densely sampled data would be better placed to support random slope specifications.

Revisions made:

"Finally, future studies with larger samples or more measurement timepoints should consider extending the current modelling approach by incorporating random slopes, allowing individual trajectories to vary across participants, or by exploring non-linear mixed models to better capture the complexity of pain trajectories over time."

16. ***Sixth, time is treated as 0, 6, 12, and 24 weeks, but the scaling of the time variable (e.g., centered, rescaled) is not reported, which affects interpretability of coefficients.***

Response:

We sincerely thank the reviewer for identifying this important issue. On re-evaluation, we confirmed that the time variable needed correction, including an error in the coding of the fourth time point. We have now corrected this throughout the manuscript and rescaled the time variable so that the coefficients reflect changes per week, thereby

improving the interpretability of the model estimates. Following this correction, we reran all relevant analyses and updated the results accordingly. This also led to revisions in the composition of the clinical compass. We greatly appreciate the reviewer's careful reading, as addressing this issue improved the accuracy and interpretability of the reported analyses. All corresponding changes have been implemented consistently throughout the revised manuscript, highlighted in yellow, and can also be traced in the GitHub repository.

17. ***Seventh, the marginal R^2 values reported in Table 3 are not clearly defined (e.g., incremental versus total explained variance), and the method used to compute them in mixed models should be specified.***

Response:

Thank you for this comment. We have clarified that the marginal R^2 represents the incremental proportion of variance explained by the fixed effects (modifiable clinical indicators) alone. This is now explicitly defined in the methods section with a reference.

Revisions made:

"*Additionally, the marginal R^2 is calculated to indicate the proportion of variance in pain intensity that can be attributed to the modifiable clinical indicators, independent of the random participant-level structure. It is derived by decomposing the total model variance into fixed-effect variance, random-effect variance, and residual variance, following the method described by Nakagawa and Schielzeth (2013). The marginal R^2 thus reflects only the contribution of the fixed effects, making it the appropriate for comparing the relative explanatory value of each clinical indicator.*"

18. ***Finally, the rationale for standardizing all indicators to z-scores for visualization, while useful for comparison, may obscure clinical meaning; no justification is provided for using sample-based standardization rather than clinically anchored thresholds.***

Response:

Thank you for this comment. Z-score standardization was chosen to enable uniform visualization of all outcomes within a single figure, allowing direct comparison of an individual's scores relative to the distribution of other participants. For most of the included metrics, which are individual items or subscales from questionnaires, no established clinically anchored thresholds exist, making sample-based standardization a pragmatic and transparent alternative.

Importantly, as discussed in the manuscript's discussion section, the proposed tool is not yet intended for clinical implementation. A causal association between the included metrics and pain intensity first needs to be established before clinical translation can

be considered. The visualization is therefore primarily intended as an exploratory and hypothesis-generating instrument, rather than a clinically actionable decision tool.

Results

19. ***In the results section, the reporting lacks sufficient quantitative detail to judge clinical relevance. Although the quadratic model is statistically superior, the practical difference between linear and quadratic fits is not quantified in terms of effect size or explained variance.***

Response:

Thank you for this comment. We respectfully note that the likelihood ratio test (LRT) reported in the results section (LR statistic = 413, df = 1, $p < 0.01$) provides a quantitative comparison between the linear and quadratic models. The LRT directly tests the improvement in model fit attributable to adding the quadratic term, and the magnitude of the test statistic (413 on 1 degree of freedom) reflects a substantial improvement in fit beyond what would be expected by chance alone.

20. ***The coefficients for time in Table 2 differ between the linear-only and quadratic models, but the interpretation of these differences is not discussed. For the significant clinical indicators, only p-values and coefficients are presented; confidence intervals and effect magnitudes are insufficiently contextualized in terms of clinical impact. There is no information on how many indicators were significant for the linear term only, quadratic term only, or both, nor how these were prioritized in the compass. The R^2 values reported appear very similar across indicators (around 0.45–0.49), raising questions about how much incremental variance each indicator contributes.***

Response:

Thank you for these comments. We address each point in turn.

Differing coefficients between models: The coefficients for time differ between the linear-only and quadratic models because they represent two distinct models, not two versions of the same estimate. When a quadratic term is added, the linear coefficient captures the instantaneous rate of change at the initial timepoint rather than the average rate of change across the full trajectory. This is a well-known property of polynomial models. Since both models serve exclusively as baseline comparisons for evaluating the addition of clinical indicators, a detailed interpretation of their differences is not clinically informative in this context. The difference in trajectories is visually represented in Figure 1.

Confidence intervals and clinical contextualization: Thank you for this comment. The primary aim of this study was to identify which modifiable clinical indicators are

significantly associated with the trajectory of pain recovery over time, rather than to precisely estimate effect magnitudes for clinical application. Since the compass is intended as an exploratory screening tool, the coefficients serve to indicate the direction of association rather than to inform clinical decision-making directly. Nevertheless, we acknowledge that confidence intervals provide useful information about the precision of the estimates, and we have therefore added confidence intervals for all fixed-effect coefficients of the significant clinical indicators to the results table.

Number of significant indicators per term: We have added a summary to the results section specifying how many indicators were significant for the linear term and the quadratic term.

"Of the N 143 clinical indicators assessed, 24 showed a significant interaction with time and 13 with quadratic time, indicating an effect on the pain trajectory (Table 3)."

Prioritisation in the compass: Indicators were prioritized for inclusion in the compass based on their marginal R^2 values, reflecting the proportion of variance in pain intensity explained by each indicator's fixed effects.

Similar R^2 values and incremental variance: We agree that the similar R^2 values across indicators do not speak to incremental variance contribution. Formally quantifying incremental variance would require a single model containing all indicators simultaneously, which falls outside the scope of this study. As stated in the introduction and discussion, the goal was not to build an optimal predictive model but to identify individual indicators independently associated with pain intensity for inclusion in a clinical compass. The compass is designed to be used at the individual level, as patients may score high on different indicators, and combining them captures clinically relevant heterogeneity that a single composite model would obscure. Further research is needed to determine how the compass can be optimally implemented in clinical practice.

21. ***Furthermore, the baseline correlations used to assess collinearity were calculated only at T0, yet some variables were time-varying; the implications of this approach are not discussed.***

Response:

We would like to clarify that correlations were calculated across all available time points for variables that were repeatedly measured. For variables assessed only at baseline, correlations could necessarily only be computed at that time point. This was already indicated in Table 1 of the original manuscript. These baseline-only variables mainly included stable demographic and clinical characteristics (e.g., age, sex, complaint duration, recurrent pain, education, and BMI), which were not expected to change during follow-up, as well as a small number of indicators that were only

conceptually relevant at study entry (e.g., “Do you experience pain in different regions?”).

22. ***The participant flow diagram is relegated to an appendix, and attrition across time points is not described in the main text, limiting transparency.***

Response:

To improve transparency, we expanded the description of participant flow in the main text, including the number of participants at each stage of inclusion and follow-up. The full flow diagram was already presented in Appendix 1. As the number of figures and tables in the main manuscript is limited, the diagram could not be included in the main paper. We believe that the revised text adequately addresses this concern.

Revisions made:

“*Of the 2,567 patients assessed for eligibility across 30 physiotherapy primary care practices, 660 provided informed consent, and 603 completed the baseline assessment and were included in this study. The main reasons for exclusion were pre-existing chronic pain, cervical spine radiculopathy, and widespread pain; 307 eligible patients declined participation. At follow-up, data were available for 449 participants at 6 weeks, 379 at 3 months, and 391 at 6 months. Only 78 participants did not complete any follow-up measurement. Participant flow is presented in Appendix 1, and the characteristics and clinical indicators of the included participants are summarized in Table 1.*”

23. ***Finally, the radar chart visualization is presented without empirical evaluation of interpretability or user comprehension, and no example is provided demonstrating how different profiles correspond to different predicted trajectories.***

Response:

Thank you for this comment. We acknowledge that the radar chart has not yet been empirically evaluated for interpretability or user comprehension. However, as this was an exploratory study, the primary aim was to develop and present the compass as a conceptual tool rather than to validate its clinical utility. Formal evaluation of the chart's interpretability and usefulness in clinical practice is the subject of ongoing future work, as discussed in the Implications for Clinical Practice section. We agree that linking individual profiles to predicted pain trajectories would strengthen the tool, and this is an important direction for subsequent validation studies.

Discussion

24. ***In the discussion, causal language is occasionally implied despite the purely observational and associational design. Statements suggesting that targeting these indicators may prevent chronic pain are speculative and should be more clearly separated from empirical findings.***

Response:
We agree that several formulations in the previous Discussion were too strong, given the observational and associational design. We have revised the wording throughout the Discussion to use more cautious, conceptually precise language, particularly where earlier phrasing could be interpreted as implying causal effects, treatment targeting, or clinical readiness. The revised text now more clearly separates the empirical findings from the more preliminary, hypothesis-generating implications for future research and practice.

Examples of revisions made:

Opening Discussion paragraph: revised to
"The clinical compass visualizes this profile to support individualized clinical reasoning and to help structure further assessment of potentially relevant domains in primary care physiotherapy."
This replaces stronger earlier wording around personalized treatment planning.

Future research: revised to
"…practice-based research… should examine whether use of the clinical compass in assessment and decision-making is associated with differences in care processes, clinical reasoning, and pain trajectories over time…"
This replaces stronger wording suggesting that targeting or modifying indicators would directly improve outcomes or prevent chronicity.

25. ***The comparison with STarT MSK does not sufficiently acknowledge that the compass has not undergone validation for predictive accuracy, calibration, or discrimination, making direct comparison premature.***

Response:
We thank the reviewer for this important observation. We agree that the previous wording suggested too direct a comparison with STarT MSK. The clinical compass was not developed as a prognostic prediction model or stratification tool, but as a profiling tool intended to visualize potentially modifiable clinical indicators and support individualized clinical reasoning. We therefore revised the Discussion to clarify that the comparison with STarT MSK is conceptual only, reflecting differences in purpose and structure rather than performance. We also added a clearer forward-looking section outlining the next steps needed to further develop the clinical compass.

Revisions made:

Comparison with previous research: revised to
"In comparison with the widely used STarT MSK, the clinical compass reflects a different conceptual approach. Whereas the STarT MSK was developed as a brief stratification tool to support risk-based care pathways, the clinical compass was developed as a

profiling tool intended to visualize potentially modifiable clinical indicators and support individualized clinical reasoning."

Also added:
"The clinical compass does not aim to classify patients into broad risk categories, but to provide a patient-specific overview of clinically relevant indicators that may guide further assessment and inform personalized care."

Implications for research: added a more explicit forward-looking paragraph on further development and validation. See our response to comment 6 of reviewer 2.

26. ***The measurement limitations of single items are acknowledged, but the implications for measurement error propagation within mixed models are not elaborated.***

Response:

Future work is required to determine the clinimetric properties of the compass tool. Without this evidence, it cannot be excluded that some included indicators may not demonstrate adequate reliability or responsiveness, potentially limiting the validity of the tool in clinical practice.

This is acknowledged as follows:

Revisions made:

"Future research should focus first on the further development and validation of the clinical compass. This includes (1) examining the content validity of the selected clinical indicators (e.g., through cognitive interviewing with patients and clinicians), (2) establishing the test–retest reliability and measurement error of the single items, (3) evaluating their responsiveness of the single questions, (4) and investigate whether targeting and modifying these items in clinical practice is associated with subsequent changes in pain trajectories."

27. ***The discussion of type I and type II error remains somewhat reassuring in tone without presenting sensitivity analyses or robustness checks to substantiate confidence in the findings.***

Response:

Thank you for this comment. We wish to clarify that the study does not claim that modifying these indicators will causally alter pain trajectories. The findings demonstrate associations between clinical indicators and pain trajectories over time, but no causal inference is made. As this was an exploratory study, the primary aim was to identify indicators related to pain trajectories, not to establish causal or temporal relationships. We agree that mediation analyses or temporal sequencing would be required to support any causal claims, and this is an important direction for future research.

Regarding treatment variation, we acknowledge that physiotherapeutic interventions were not standardized across practitioners, which represents a potential source of confounding. This has been added to the limitations section. Future studies should consider controlling for treatment type and intensity to better isolate the contribution of the identified indicators to pain trajectory outcomes.

28. ***Moreover, the clinical implications assume that modifying these indicators will alter pain trajectories, yet no mediation or temporal sequencing analyses were performed to support this assumption. The potential confounding effect of treatment variation across physiotherapists and practices is not addressed, even though therapeutic interventions were not standardized and could influence pain trajectories independently of baseline indicators.***

Response:

Thank you for this important comment. We agree that our findings do not demonstrate that changing the identified indicators will necessarily alter pain trajectories. The purpose of the clinical compass is not to establish causal effects, but to visualize clinical indicators that may reflect relevant underlying biopsychosocial processes and thereby support individualized clinical reasoning. We have therefore revised the manuscript to frame the clinical implications more cautiously and to avoid causal interpretation.

We also acknowledge that treatment variation across physiotherapists and practices may have influenced pain trajectories independently of the identified indicators. Participants received non-standardized usual care in routine primary care practice, and this variation was not accounted for in the present analyses. We have now added this more explicitly as a limitation in the manuscript.

Revisions made:

Discussion, Methodological limitations:

"By making such clinical indicators explicit, the clinical compass may support more informed and structured individualized clinical reasoning. However, the clinical compass should be understood as a profiling tool rather than as evidence that changing the identified indicators will necessarily alter pain trajectories. The present analyses were associative in nature and did not evaluate mediation, temporal causal ordering, or treatment effects. Therefore, the compass is intended to help clinicians identify potentially relevant domains for further assessment, rather than to imply that modifying an identified indicator will automatically improve outcomes."

"In addition, participants received usual physiotherapy care in routine primary care practice, and treatment was not standardized across physiotherapists or practices.

Such variation may have influenced pain trajectories independently of the indicators included in the clinical compass and was not accounted for in the present analyses."

Discussion, Implications for practice:

"Future implementation efforts would likely require targeted training to help therapists interpret the clinical compass appropriately, integrate it into routine assessment, and strengthen therapists' skills to address potential modifiable factors."

Conclusion

29. ***In the conclusion, the statement that the compass provides a "clinically feasible method" is premature in the absence of usability testing, implementation research, and external validation. The conclusion should more clearly delimit the scope of inference to exploratory identification of candidate indicators rather than implying readiness for clinical deployment.***

Response:
We agree that this wording overstated the readiness of the clinical compass for clinical use. We removed the phrase *"clinically feasible method"* and revised the Conclusion to frame the findings more clearly as exploratory and prototype-based.

30. ***It would also be important to emphasize that the compass reflects associations with pain intensity trajectories in a specific Dutch primary care context and may not generalize without replication.***

Response:
We agree and made this contextual scope more explicit in the revised Conclusion, see our response under comment 31.

31. ***Finally, the conclusion does not adequately reflect the substantial statistical uncertainty introduced by multiple testing and univariable screening.***

Response:
We agree that the exploratory nature and statistical uncertainty should be more clearly reflected. Rather than overloading the Conclusion with methodological detail, we revised it to adopt a more cautious formulation and expanded the Discussion to address uncertainty related to multiple testing and univariable screening more explicitly.

Revisions made:

Conclusion: revised to
"*This study identified several biological, psychological, and behavioral clinical indicators associated with change in pain intensity over time in patients with nonspecific neck pain. The indicators were integrated into a 22-item prototype clinical*

compass to visualize patient-specific profiles and support prognostic reasoning in primary care. The findings are exploratory, reflect associations observed in a Dutch primary care physiotherapy context, and require replication, external validation, and evaluation of usability and clinical impact before clinical implementation."

Reviewer: 2
Mr. Musa Danazumi, La Trobe University Discipline of Physiotherapy
Comments to the Author:
Overall Recommendation: Minor Revision
This manuscript presents an innovative secondary analysis of a large prospective cohort exploring modifiable clinical indicators associated with pain intensity trajectories in individuals with (sub)acute non-specific neck pain. The study is clinically relevant, methodologically sound, and aligns well with contemporary efforts to operationalise personalised physiotherapy.
The longitudinal design, repeated measurements, biopsychosocial breadth of indicators, and use of growth-curve modelling are clear strengths. The development of the "clinical compass" as a translational tool is particularly novel and represents an important step toward bridging research and clinical reasoning.
Overall, the manuscript is of high quality. I offer several suggestions that may help strengthen clarity and interpretability.

Major Suggestions

1. ***Clarification of Exploratory Nature; Given the large number of clinical indicators evaluated, it may be helpful to explicitly frame the analysis as exploratory and hypothesis-generating. While adjustment for multiple testing is not always required in exploratory analyses, a brief justification of this decision would improve transparency. This would strengthen the methodological clarity without altering the study's conclusions.***

Response:

Thank you for your suggestion. We emphasized the exploratory nature of this study and added the following sentence to the methods section.

Revisions made:

"No correction for multiple testing was applied, since all the included variables were pre-selected based on theoretical and clinical relevance to pain trajectories, which constrains the risk of spurious findings and the analysis was mainly exploratory."

2. ***Modelling Strategy Transparency***
Each modifiable indicator appears to have been added separately to the growth model. Clarifying that this represents a screening or effect-modifier exploration approach (rather than a fully adjusted multivariable prediction model) would help readers interpret the findings appropriately.

It may also be useful to briefly acknowledge that interrelationships between predictors were not modelled simultaneously.

Response:

Thank you for this comment. We agree that this clarification is important for correct interpretation of the findings. Each clinical indicator was added separately to the growth model, representing a screening approach aimed at identifying which individual indicators are independently associated with pain trajectories, rather than constructing a fully adjusted multivariable prediction model. Consequently, interrelationships between predictors were not modelled simultaneously, meaning that shared variance between indicators is not accounted for. We have explicitly acknowledged this in the methods sections to ensure readers interpret the findings as an exploratory screening approach rather than a predictive modelling exercise.

To further support transparency and reproducibility, we also now explicitly state in the manuscript that the complete dataset and Python script used in this study are available on GitHub.

Revisions made:

"This approach was used as an exploratory screening strategy aimed at identifying which individual indicators are independently associated with pain trajectories, rather than constructing a fully adjusted multivariable prediction model. Consequently, interrelationships between predictors were not modelled simultaneously."

And for further support transparency:

"The complete dataset and Python script used in this study are available on GitHub at https://github.com/RichardFel/Paincare [10]."

3. ***Time-Varying Indicators***
   Some indicators were measured repeatedly. A short clarification on whether these were treated as time-varying covariates, and how temporal ordering was handled, would enhance methodological transparency.
   Even a concise statement in the Methods would be sufficient.

Response:

Thank you for your suggestion we added the following line to the results to clarify that these were treated as time varying covariates.

Revisions made:

"This approach was used as an exploratory screening strategy aimed at identifying which individual indicators are independently associated with pain trajectories, rather than constructing a fully adjusted multivariable prediction model. Consequently, interrelationships between predictors were not modelled simultaneously."

4. ***Interpretation of Marginal R^2***
   The use of marginal R^2 to rank indicator relevance is helpful. Providing a brief description of how R^2 was calculated and clarifying that it reflects variance explained within the modelling framework would improve interpretability.

Response:

Thank you for your suggestion. We added a description of how the R^2 was calculated.

Revisions made:

"Additionally, the marginal R^2 is calculated to indicate the proportion of variance in pain intensity that can be attributed to the modifiable clinical indicators, independent of the random participant-level structure. It is derived by decomposing total model variance into fixed-effect variance, random-effect variance, and residual variance, following the method described by Nakagawa and Schielzeth (2013). The marginal R^2 thus reflects only the contribution of the fixed effects, making it the appropriate for comparing the relative explanatory value of each clinical indicator."

5. ***Clinical Compass Interpretation***
   The clinical compass is a highly innovative feature of the manuscript. Since it is based on standardized z-scores relative to the cohort mean, it may be helpful to emphasize in the Discussion that this tool currently supports structured clinical reasoning rather than providing prescriptive thresholds.

Response:
We thank the reviewer for this helpful suggestion. We agree that the current version of the clinical compass should be framed as a tool to support structured clinical reasoning rather than as an instrument with prescriptive thresholds. We revised the Discussion accordingly to clarify that the current z-score visualization is descriptive and intended to highlight domains for further reflection or assessment, while clinically meaningful thresholds remain to be established in future research.

Revisions made:

Discussion: added
"Finally, the current version of the clinical compass presents results in a radar chart using standardized scores (z-scores) to show how an individual patient deviates from the cohort mean. At this stage, these deviations are intended to support structured clinical reasoning rather than to function as prescriptive thresholds. The visualized profile may help identify domains for further assessment, but does not yet indicate whether observed differences are clinically meaningful. Future research should therefore investigate how the interpretability and utility of the clinical compass can be strengthened, including the potential development of clinically relevant reference values."

6. ***A short forward-looking statement about future validation would further strengthen the translational message.***

Response:
We thank the reviewer for this helpful suggestion. While the previous version of the Discussion already contained several forward-looking remarks, they were scattered throughout the text. To improve clarity, we have now made the implications for research more explicit and consolidated them into a clearer forward-looking section. This section outlines the key next steps for further development of the clinical compass, including evaluation of content validity, reliability, responsiveness, usability, interpretability, and its potential value in clinical practice.

Revisions made:

Implications for research: added and consolidated the following paragraph:
"Future research should focus first on the further development and validation of the clinical compass. This includes (1) examining the content validity of the selected clinical indicators (e.g., through cognitive interviewing with patients), (2) establishing the test–retest reliability and measurement error of the single items, and (3) evaluating their responsiveness. In addition, studies are needed to assess the usability, interpretability, and clinical utility of the clinical compass as a tool to support structured assessment and individualized clinical reasoning. Finally, practice-based research, such as pragmatic prospective studies or single-case experimental designs, should examine whether use of the clinical compass in assessment and decision-making is associated with differences in care processes, clinical reasoning, and pain trajectories over time across independent samples and settings."

7. ***Minor Suggestions***
   • Brief clarification of missing data assumptions (e.g., MAR in LMM).

Response

We expanded the method section to include the assumptions. See also our response to comment 11 of reviewer 1.

Revision made:

"They also allow the inclusion of all available repeated observations, so that participants with incomplete follow-up data can be retained under the assumption that data are missing at random."

8. ***• Report attrition at each time point***

Response

This point has been addressed in our response to comment 20 by Reviewer 1. In brief, we expanded the description of participant flow in the main text, including attrition at

each follow-up time point, while the full participant flow diagram remains available in Appendix 1.

Reviewer: 1
If you have selected ‘Yes’ above, please provide details of any competing interests.: NO

Reviewer: 2
If you have selected ‘Yes’ above, please provide details of any competing interests.: N/A
